# Fecal microbiomes from healthy adult consumers of fruits and vegetables exhibit fiber- and donor-specific fermentation: “5 a day” is not enough†

**DOI:** 10.1039/d5fo00947b

**Published:** 2025-09-08

**Authors:** Sarah E. Blecksmith, Karen M. Kalanetra, Cheng-Yu Weng, Christopher Suarez, Irnayuli R. Sitepu, Yirui Tang, Shawn Ehlers Cheang, Sophia Jiang, Karina Cernioglo, Karla Damian-Medina, Jennifer T. Smilowitz, Carlito B. Lebrilla, David A. Mills, Danielle G. Lemay

**Affiliations:** a Department of Food Science and Technology, University of California, Davis Davis California USA damills@ucdavis.edu; b Department of Chemistry, University of California, Davis Davis California USA; c Department of Nutrition, University of California, Davis Davis California USA danielle.lemay@usda.gov; d USDA-ARS Western Human Nutrition Research Center Davis California USA

## Abstract

To determine the fermentation capacity of gut microbiomes with diverse plant carbohydrate active enzyme (CAZyme) repertoires, we collected fecal samples from 18 healthy adults who reported consuming at least 5 different fruits and vegetables daily and conducted shotgun metagenome analysis. Five fecal samples with the most diverse CAZymes were then fermented *in vitro* with 7 different fibers selected for their unique monosaccharide profiles—banana, kale,13-bean soup, flax, coconut flour, MS Prebiotic (resistant starch) and Sunfiber (guar gum)—for 72 hours. Samples were collected at 4 timepoints for 16S sequencing, and pH, SCFAs, and monosaccharide measurements. The largest changes in pH, microbial diversity, monosaccharides, and short chain fatty acids (SCFAs) occurred in the first 24 hours of fermentation. SCFA production was highest with flax and lowest with coconut flour. Fermentation patterns ranged from little change to primary degradation (liberated monosaccharides) to robust production of SCFAs. Abundance of *Bifidobacteriaceae*, *Butyricicoccaceae*, and *Ruminococcaceae* correlated with the highest fermentation, *Clostridiaceae*, *Enterococcaceae*, and *Eggerthellaceae* with the lowest. Samples from three of the participants were more responsive than the other two. The donor-specific and fiber-specific responses seen in our study indicate that dietary guidance to consume 5 servings of fruits and vegetables per day may not be enough to ensure that our gut microbiota is capable of unlocking all of fiber's benefits.

## Abstract 2

The microbiomes of regular consumers of fruits and vegetables show variable ability to degrade specific fiber and produce SCFA.

## Graphical abstract

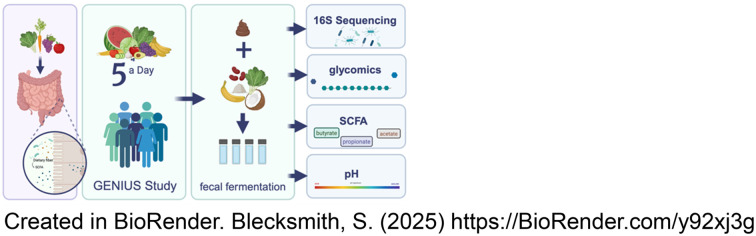

## Introduction

The Western diet is characterized by ultra-processed foods and low intake of dietary fiber. The 2020–2025 Dietary Guidelines for Americans (DGA) recommend that adults consume 14 grams of fiber per 1000 calories but more than 90% of American women and 97% of American men do not meet this threshold.^1^ The DGA recommendation for fruit and vegetable intake is 2.25 cup equivalents per 1000 calories per day, with only 14% of adults fully meeting this goal.^2^ The DGA recommendations for fiber are based on blood cholesterol-lowering effects, not fermentative capacity. In 1988, the California Department of Health Services began the “5 a Day – For Better Health” campaign to educate the public on the health benefits of consuming 5 servings of fruits and vegetables daily.^3^ The campaign was later implemented nationwide by the National Cancer Institute and the Produce for Better Health Foundation. This level of fruit and vegetable intake was found to reduce overall risk of dying from cancer, cardiovascular disease and respiratory disease.^4^

Dietary fiber is considered a crucial component of nutrition in the human diet, and primarily recognized for its function in promoting digestive health and maintaining bowel regularity.^5^ Beyond its structural and digestive benefits, dietary fiber is resistant to digestion by human enzymes and can reach colon where it significantly influences the composition and functionality of the gut microbiota.^6^

At the simplest level, fiber is often characterized as “soluble and insoluble”^7^ with soluble fiber being fermented by the gut microbiota (*e.g.* gums, pectins, mucilages, inulin, beta-glucans, arabinoxylans and oligosaccharides), while insoluble fiber (*e.g.* starch, cellulose, lignan, and hemicellulose^8^) being much less fermentable and providing bulk to stool. However, these designations are misleading and incomplete. All fiber can be digested (at least in part) given the proper enzymatic repertoire in the bacterial community. Thus, when metabolized by gut bacteria, dietary fibers serve as prebiotic, selectively stimulating the growth of beneficial bacterial communities.^9^ The interactions between dietary fiber intake, microbial composition, and host health suggest that dietary fiber is essential in shaping a balanced gut microbiota, with potential implications for reducing the risk of chronic diseases such as obesity, diabetes, and colorectal cancer.^10^ Therefore, exploring the types, quantities, and sources of dietary fiber offers valuable insights into modulating gut microbiota composition towards the ultimate goal of overall health enhancement.^11^

Diets low in fiber cause a loss of microbial species over generations in mice and a loss of fiber-degrading capacity.^12^ Microbes utilize carbohydrate active enzymes (CAZymes) to degrade the glycans that escape digestion and absorption in the small intestine. Differences in CAZyme repertoire have been seen by diet between: Hadza hunter-gatherers and Americans; Hadza in wet and dry seasons, when diet changes;^13^ and participants’ habitual diet and a prescribed low fiber homogenous diet.^14^ Industrialized people have lost cellulose-degrading ability in their microbiomes as compared to rural people, hunter-gatherers, paleofeces samples, and non-human primates.^15^

Low fiber diets are also linked to lower levels of important metabolites produced by the gut microbiota such as short chain fatty acids (SCFAs). These compounds, primarily acetate, propionate, and butyrate, have been associated with several positive health outcomes, including reducing inflammation in the gut and systemically, strengthening gut barrier function, improving blood sugar regulation and increasing satiety.^16–19^ However, because most SCFAs produced in the gut are absorbed and metabolized by the intestinal epithelium it can be difficult to connect fecal SCFA levels with health outcomes in humans.^20^ Many studies are performed *in vitro* or in animal models to understand mechanisms.

Compounding the challenges in studying fiber intake and the gut microbiota is the rather glaring lack of knowledge on the precise chemical structure(s) of the consumed fibers. Carbohydrates have traditionally been determined in foods by gravimetric analysis, rather than directly measured.^21^ Thus, historically, little was known about their monosaccharide composition and linkages. However, this is beginning to change with the development of comprehensive glycomic platforms^22^ and the publishing of the Davis Food Glycopedia,^23^ an online database reporting the comprehensive monosaccharide composition of hundreds of foods. Glycan analysis can similarly be applied to fecal samples,^24,25^ or after *in vitro* fermentation,^26^ enabling a comparison to the carbohydrates entering the system to determine what was fermented.

With detailed molecular description of carbohydrate compositions, the impact of dietary fibers on the microbiota and SCFA production can be studied *with much higher resolution*. Therefore, we sought to investigate variation in fermentation of fiber types, chosen for their structural diversity, by inoculum derived from human participants with a particular baseline diet—those who consume at least five fruits and vegetables per day. We recruited 21 regular fruit and vegetable consumers to donate stool. Participant fecal metagenomes were sequenced and mapped to the CAZy database. We chose stool samples from the 5 participants with the highest plant CAZyme diversity for fecal fermentations. Five foods and two fiber supplements were selected for fermentation based on their monosaccharide diversity from the Davis Food Glycopedia.^23^ With the diverse CAZyme stools, we performed *in vitro* fecal fermentations over 72 hours and measured free and total monosaccharides, SCFA production, pH, and microbial composition with 16S sequencing. We hypothesized that all fibers would be fermentable with differences, by fiber type, in timing of SCFA production.

## Materials and methods

### Study population

Between January 2021 and April 2021, healthy adults that consumed at least 5 fruits and vegetables per day and lived within Yolo and Sacramento Counties in California were recruited to enroll in the Gut Microbiome and Carbohydrate Function of Healthy Adults (GENIUS) Study. Inclusion criteria for study participation were as follows: healthy adults aged 18–65 years, with normal stool frequency defined as at least three times per week or up to three times per day, an average stool consistency of type 3, 4, or 5 stool as defined by the Bristol stool scale.^27^ Exclusion criteria for study participation were as follows: history of any chronic metabolic, inflammatory, immune, endocrine, or infectious diseases; obesity; food allergies; eating disorders; chronic constipation; chronic diarrhea; bariatric or gastric surgery; frequent cannabis use; frequent illicit drug use; excessive alcohol consumption; stomach ulcers or *H. pylori* infection within the past 12 months; antibiotic use within the past 6 months; vaccine administration within the past 4 weeks; use of restrictive diets resulting in weight loss greater than 10% of body weight within the past 12 months; current tobacco use or tobacco use within the past 12 months; probiotic supplement use or consumption of food products containing probiotics including kefir, kombucha, and yogurt within the past 8 weeks; consumption of fermented foods within the past week, and use of medications that could impact the gut microbiota.

### Study design

The GENIUS study was an observational, prospective trial that lasted up to 7-weeks or until participants collected a total of at least 300 grams of stool. After meeting all study criteria, individuals provided written, informed consent. The University of California Davis Institutional Review Board approved all aspects of the study (IRB #: 1600677).

The study period consisted of a one-week lead-in period, during which participants were asked to complete daily logs about their health and dietary intake of confounding variables, followed by an up to 7-week stool collection period during which they continued completing daily logs and collected a total of at least 300 grams of stool. During the first week of the study participants were asked to complete a health history questionnaire to gather data about their demographics and general health, lifestyle, and gut history. Participants also completed the web-based Block 2014 Food Frequency Questionnaire that contains 127 food and beverage line items, portion size pictures, and additional questions to adjust for fat, protein, carbohydrate, sugar, and whole grain content and a physical activity screener (Nutrition Quest, Berkeley, CA, USA). Throughout the study participants completed two daily logs including a Daily Health Log to report their number of daily stools and the consistency of their first stool of the day, per the Bristol Stool Scale, illnesses, and intake of medications and a Daily Intake Log to report the consumption of confounding variables of the gut microbiota. Following each stool sample collection participants were asked to complete a 24-hour dietary recall.

To reduce confounding variables participants were instructed to avoid consuming probiotic supplements, food products containing probiotics and fermented foods such as yogurt, kefir, kimchi, kombucha, miso, and sauerkraut throughout the study period.

### Sample collection

Participants were asked to collect a total of at least 300 grams of stool using the Fisherbrand™ Commode Specimen Collection System. Participants were instructed to collect stool alone, excluding urine, and female participants were asked to collect stool samples when they were not menstruating. Following each stool sample collection, participants weighed the collected stool. They were asked to collect additional stool samples until the total amount of stool collected was at least 300 grams. Collected samples were immediately stored in participants’ home freezers until they were transported to the laboratory on frozen ice packs, after which they were stored at −80 °C until processed. Studies have shown that initial home freezer storage of stool samples followed by storage at −80 °C produces stable results for metabolomics^28^ and shotgun metagenomics.^29^

### Calculation of dietary indices

Healthy Eating Index (HEI) 2020 scores for the FFQ were calculated using R and macros provided by Zhan *et al.*^30^ Dietary fiber intake, fruit and vegetable intake, and soluble and insoluble fiber were provided by NutritionQuest who sources the Block FFQ.

### DNA extraction and shotgun metagenomic sequencing

DNA was extracted from fecal samples as described previously [1]. Briefly, the ZymoBiomics DNA miniprep kit (Zymo Research) was used to isolate DNA from 100 mg homogenized stool. DNA quality was assessed with Nanodrop ND-1000 Spectrophotometer (ThermoFisher) with the majority (>95%) of samples having *A*_260/280_ and *A*_260/230_ ratios above 1.80. Before library prep, the DNA samples were confirmed to be intact and free of RNA with gel electrophoresis. The Qubit double-stranded DNA (dsDNA) broad-range assay (ThermoFisher) was used to quantify DNA and samples were diluted to 100 ng μL^−1^.

As described previously,^31^ the DNA Technologies and Expression Analysis Core Laboratory at the University of California at Davis, Genome and Biomedical Sciences Facility performed whole-genome shotgun sequencing library preparation, quality control, quantification and pooling. DNA was sequenced on an Illumina NovaSeq in 2 × 150 bp format.

### Metagenomics analysis

Bioinformatics processing of sequencing reads was conducted as previously detailed.^31^ In short, reads aligning to the human genome were removed with BMTagger^32^ aligning to human genome version GRCh_3_8.p13.^33^ Then, Trimmomatic version 33^34^ was used to remove adapters and trim paired-end reads with a sliding window of 4 bp, a minimum average quality of 15, and a minimum length of 99 bp as previously described.^35^ Afterward, FastUniq version 1.1^36^ with default settings was used to remove duplicate reads. FLASH version 1.2.11^37^ was used to merge paired-end reads with an overlapping read length range between 10 bp to 100 bp and a mismatch ratio of 0.1. The merged reads were aligned to the CAZy database^38^ using DIAMOND^39^ in blastx mode. MicrobeCensus^40^ was used to calculate the average genome size, total reads, and genome equivalents. These were used to create a RPKG (reads per kilobase per genome equivalent) normalized count table. Microbial taxonomy was profiled using MetaPhlAn4.^41^

### CAZymes and sample selection

The enzymes that degrade carbohydrates, known as carbohydrate active enzymes (CAZymes), are classified and categorized in the continuously updated CAZy database (https://www.cazy.org).^38^ The database has defined six enzyme classes: glycoside hydrolases, glycosyl transferases, polysaccharide lyases, carbohydrate esterases, carbohydrate-binding modules and auxiliary activities. The enzyme classes are further broken down into families. For the study of carbohydrate degradation, the glycoside hydrolases (GH) and polysaccharide lyases (PL) are the classes of interest. Thus, in the present study, we investigated CAZyme diversity of GH and PLs, specifically, and considered individual CAZyme classes.

After mapping to the CAZy database, the normalized counts for CAZyme genes were aggregated into CAZyme families and subfamilies with a custom script. CAZyme family substrates were annotated using the scheme from Smits *et al.*^13^ Plant unique CAZyme families were those families with plant substrates that were not in any other substrate categories. Shannon and Chao1 diversity and the observed count of plant unique CAZyme families were calculated with R package phyloseq.^42^

### Fecal homogenization

To prepare the feces for fecal fermentations and for long-term storage the collected stool was homogenized and mixed with glycerol : PBS solution as follows. After collection, a portion of participants’ stool was thawed on ice and homogenized. In a Coy anaerobic chamber with anaerobic atmospheric gas mix of 5% carbon dioxide, 5% hydrogen and 90% nitrogen gas, 4 g fecal aliquots were weighed out and transferred to 15 ml sterile falcon tubes and mixed by vortexing with the PBS : glycerol solution so that the final glycerol concentration was 20%. The aliquots were stored at −80 °C until further use.

### Preparation of foods and fibers

*In vitro* digestion of seven foods (100 g banana, 100 g kale, 100 g Bob's Red Mill 13 Bean Soup Mix, 40 g flax meal, 30 g coconut flour, 35 g Sunfiber, and 50 g MS Prebiotic) were performed with the INFOGEST 2.0 protocol.^43^ The goal was to have enough digested fiber to include in each fecal fermentation a final 1% carbohydrate concentration. Each food was mixed with an equal volume of simulated salivary fluid (the final concentration of salivary amylase [Megazyme E-PAANA] was 75 IU ml^−1^). During the simulated gastric phase, the final concentration of pepsin (Sigma, P6887) was 2000 U ml^−1^. No gastric lipase was added. During the simulated intestinal phase, the final concentration of pancreatin from porcine pancreas (Sigma, P7545) was 100 U mL^−1^, and that of bile extract (Sigma, B3883) was 10 mmol mL^−1^. The final volume of each digested product was 800 ml and was immediately frozen at −80 °C to halt enzymatic activity. The digest was then freeze dried (Harvest Right, HR7000-M) and the freeze-dried digest reconstituted to 300 ml with deionized (DI) water. To remove monosaccharides and salts, the reconstituted freeze-dried digest was dialyzed in 2000 NMWCO dialysis tubes (Sigma, D7884) for 4 days at 4 °C against DI water, the water being changed for fresh DI water 3 times on day 1 and twice a day on days 2 to 4. The digested and dialyzed product was freeze dried (Harvest Right, HR7000-M), homogenized in a coffee grinder, weighed and stored in 50 ml falcon tubes in a desiccator at −20 °C. All lyophilized powders were stored in sealed secondary containers with desiccant prior to anthrone assays.

Anthrone assays were performed to determine the total carbohydrate content of the lyophilized powders. A serial dilution of fibers and amylopectin standards were prepared at 0.75 mg mL^−1^, 0.6 mg mL^−1^, 0.5 mg mL^−1^, 0.4 mg mL^−1^, 0.25 mg mL^−1^, and 0.1 mg mL^−1^. Seventy ul of serially diluted fibers or amylopectin was combined with 140 μL of 2 mg mL^−1^ anthrone in 6 M sulfuric acid in 0.5 mL strip tubes. Reactions were run in duplicate. The strip tubes were centrifuged and run in a Thermocycler at 90 °C for 11 min followed by 20 °C for 8 min. Samples were transferred to 96-well plates, and fluorescence data were obtained with a plate reader. The total carbohydrate content of the lyophilized powder was then calculated. The complete fiber processing procedure is illustrated in Fig. S1.†

### Fecal fermentations

The fermentation media composition was based on that of Walker and coworkers,^44^ and was composed by combining the following components per liter DI water: biotin, 100 μg; CaCl_2_·2H_2_O, 20 mg; FeSO4·7H_2_O, 5.4 mg; l-cysteine HCl, 500 mg; bile salts, 50 mg; Bacto Casitone, 1 g; Bacto Proteose peptone no. 3, 670 mg; NaCl, 4.83 g; MgSO_4_·7H_2_O, 500 mg; hemin, 10 mg; K_2_HPO_4_, 5 g; KH_2_PO_4_, 3.19 g; NaHCO_3_, 1.93 g; Na_2_CO_3_, 2.33 g; Tween-80, 2.0 g; and MES·H_2_O, 9.76 g; ZnSO_4_·7H_2_O, 20 μg; MnCl_2_ 7H_2_O, 6 μg; EDTA 1.0 mg; boric acid, 60 μg; CoCl_2_·6H_2_O, 40 μg; CuCl_2_·2H_2_O, 2 μg; NiCl_2_·6H_2_O, 4 μg; NaMoO_4_·2H_2_O, 6 μg; menadione, 1 μg; *para*-aminobenzoic acid, 0.5 μg; pantothenate, 10 μg; nicotinamide, 5 μg; cyanocobalamin, 0.5 μg; and thiamine-HCl, 4 mg, 3.5 ml 100% ethanol. Stock solutions were filter sterilized with a 0.22 μm polyethersulfone filter. The prepared fibers were the only carbon source in the custom media.

Each of the seven processed fibers were used to carry out a batch fecal fermentation experiment with the 5 participants so that there were 7 separate batch experiments in total. For each experiment, the homogenized fecal inoculum of the 5 participants was prepared by thawing the participant's stool on ice in the anaerobic chamber to prevent microbial growth. The fecal tubes were vortexed for 5 minutes and spun in a tabletop centrifuge at 4 °C at 200*g* for 10 minutes to remove any solid food or large particles in the feces. One milliliter of fecal slurry was added to 15 ml of medium and fiber for a total of 16 ml fecal fermentation.

The batch fecal fermentation method was based on a protocol described previously,^45^ but performed here with human stool and not using the minibioractor array. Instead, the fermentations were carried out in triplicate in an anaerobic environment (Coy anaerobic chamber with anaerobic atmospheric gas mix of 5% carbon dioxide, 5% hydrogen and 90% nitrogen gas) at 37 °C and included controls of media, stool and media, and fiber and media for each participant. Each 16 ml fermentation was carried out in a 30 ml sterile capped glass tube on a magnetic stir plate and constantly mixed during the incubation. Samples were collected at time 0, and days 1, 2 and 3 for pH, DNA extraction, monosaccharide compositions, and SCFA measurements. At each time point samples were collected with sterile, disposable serological pipets and transferred to sterile 1.5 ml microcentrifuge or 2 ml screw cap collection tubes as such: 0.5 ml for each, separately, pH, monosaccharide composition, and SCFA measurements and 1.0 ml for DNA extraction, stored in DNA/RNA Shield (Zymo Research, Irvine, CA) and stored at −20 °C or −80 °C prior to analysis.

### 16S sequencing

The Zymobiomics 96 MagBead DNA Kit (Zymo Research, Irvine, CA) was used to extract genomic DNA with a Kingfisher Flex (Thermo Fisher Scientific, Waltham, MA). The V4 region of the 16S rRNA gene was PCR amplified in triplicate with primers F515 and R806 as previously described.^46^ Amplicons were verified by gel electrophoresis then combined, purified, and sent to the UC Davis Genome Center for library preparation and high throughput 250 bp paired end sequencing with an Illumina MiSeq.

Resulting sequencing raw data was demultiplexed with sabre^47^ and imported into the QIIME2 software package (version QIIME2-2022.11).^48,49^ Trimmed reads were quality filtered and processed with DADA2^50^ After filtering, taxonomy was assigned using the 99% SILVA naive Bayes classifier in QIIME2-2022.11.^51^

### Quantitation of total monosaccharide composition

The monosaccharide analysis of bioreactor sample was modified from previously reported methods.^22,52^ Briefly, 10 μL aliquots from homogenized stock solutions were transferred to a 96-well plate (2 mL wells). Each sample was hydrolyzed with hard acid (4 M trifluoracetic acid for 1 hour at 121 °C) after which the reaction was quenched by addition of 855 μL of nanopure water. Following hydrolysis, 10 μL aliquots of hydrolyzed sample and 50 μL of an external calibration curve of 14 monosaccharide standards with concentrations ranging from 0.001–100 μg mL^−1^ were derivatized with 0.2 M 1-phenyl-3-methyl-5-pyrazolone (PMP) in 1 : 1 (v/v) methanol (MeOH) and 28% NH_4_OH for 30 minutes at 70 °C. After the reaction was complete, derivatized samples were dried overnight by vacuum centrifugation, reconstituted in nanopure water, and excess PMP was removed by chloroform extraction. A 1 μL aliquot of the aqueous layer was injected into an Agilent 1290 Infinity II UHPLC system equipped with an Agilent Poroshell HPH-C18 column (2.1 × 50 mm, 1.9 μm) and guard in 2 minutes with an isocratic elution of 12% solvent B. Solvent A consisted of 25 mM ammonium acetate adjusted to pH 8.2 using concentrated ammonia solution and solvent B consisted of 95% acetonitrile in water. The separated monosaccharides were then detected by an Agilent 6495A QqQ-MS operated in multiple reaction monitoring (MRM) mode and quantitation of monosaccharides was achieved by comparison to the external calibration curve.

### Quantitation of free monosaccharide composition

The free monosaccharide content of bioreactor samples was obtained by modifying the total monosaccharide protocol. Briefly, each homogenized stock solution was centrifuged at 10 000 rpm for 15 minutes. 10 μL aliquots of the sample's supernatant were transferred to a 96-well plate (2 mL wells) as well as 50 μL of an external calibration curve of 14 monosaccharides with concentrations ranging from 0.001–100 μg mL^−1^. Each unhydrolyzed sample was then derivatized by PMP according to the protocol above for total monosaccharide analysis. Excess PMP was removed by chloroform extraction and 1 μL aliquot of the aqueous layer was subjected to UPLC-QqQ MS analysis as described above for the analysis of total monosaccharide composition.

### Quantitation of short-chain fatty acid composition

The short-chain fatty acid and lactate content of bioreactor samples was quantified by adapting previously reported methods.^53^ In brief, 200 μL of acetonitrile (ACN) and 100 μL of derivatization reagent containing 20 mM triphenylphosphine (TPP), 20 mM dipyridyl disulfide (DPDS), and 20 mM 2-picolylamine (2-PA) in ACN were plated in a 1 mL 96-well plate before adding samples. Then, 10 μL of each sample was added to the reaction solution, along with pooled standard solutions consisting of 18 carboxylic acid metabolites prepared in MeOH and serially diluted to different concentrations ranging from 0.001 to 500 μg mL^−1^. An internal standard mixture containing 100 μg mL^−1^ of d^4^-acetic acid, 50 μg mL^−1^ of d^2^-indolepropionic acid, and 10 μg mL^−1^ of 2-ethylbutyric acid was spiked into all standards and samples at a ratio of 1 : 10 (v/v) before plating. The plate was then sealed, and the sample was incubated at 60 °C for 10 minutes. The whole procedure was conducted in a 4 °C cold room to reduce the evaporation of volatile analytes. Following completion of the reaction, the derivatized samples were dried in a miVac concentrator. The dried samples were reconstituted in 500 μL of 50% MeOH before instrumental analysis. 1 μL of derivatized sample was analyzed on an Agilent 6495B QqQ MS coupled to an Agilent 1290 Infinity II UHPLC and equipped with an Agilent Poroshell 120 EC-C18 column (2.1 × 100 mm, 1.9 μm particle size). Aqueous mobile phase A consisted of 100% nanopure water. Organic mobile phase B consisted of a 1 : 1 (v/v) ACN/isopropyl alcohol (IPA) mixture. The following binary gradient was used: 0.00–1.00 minutes, 5.00% B; 1.00–10.00 minutes, 5.00–20.00% B; 10.00–11.00 minutes, 20.00% B; 11.00–15.00 minutes, 20.00–60.00% B; and 15.00–16.00 minutes, 60.00–5.00% B. The mobile phase flow rate was 0.45 mL min^−1^, and the column temperature was set to 45 °C. Mass spectrometry data was collected in the dMRM mode. SCFA measurements are missing for one fermentation replicate of Participant 3 with flax.

### Analysis

Analyses were performed in R with custom scripts (https://github.com/sblecksmith/genius_project). Plant CAZyme diversity from starting shotgun metagenomes was calculated with R package phyloseq 1.44.0.^42^ After removing non-bacterial phyla, Shannon diversity and Pielou's evenness of the microbial taxa of the fermentations were calculated with phyloseq and the R package microbiome 1.22.0,^54^ respectively. SOM was made with R package kohonen 3.0.12.^55^ PERMANOVA was conducted with the R package vegan 2.6–6.1.^56^ The correlation matrix was made with the R package microViz 0.12.0, testing for correlation with Spearman's Rank test and p values less than 0.05 and FDR adjusted *p* values less than 0.05 labeled separately.^57^ Changes in Shannon diversity, Pielou's evenness, SCFA production and free and total monosaccharides were calculated as the difference between the 24 hours sample and the 0 hour sample. These differences were compared using Wilcoxon signed-rank tests with Bonferroni correction. Adjusted *p*-values <0.05 were considered statistically significant.

## Results

### Study participants

Fifty-two adults were screened for eligibility to participate in the study. Twenty-one adults provided informed consent and were enrolled in the study. Stool samples were collected from nineteen participants; however, one participant's collected stool was excluded from analysis due to insufficient DNA yield/quality. Thus, a total of eighteen stool samples from eighteen participants were analyzed. Sixty-one percent of participants were female and 38% were male with ages ranging from 20–47 years with a mean of 30.6 years, and a BMI ranging from 18.4–32.3 kg m^−2^ with a mean of 23.7 kg m^−2^. Participant demographic details are given in Table S1.† Table S2† provides general health history information. Additional dietary details are given in Tables S3–S9.†

### Participant diets rich in fruits, vegetables, whole grains, and fiber

Participants were recruited for the study if they reported consumption of at least 5 servings of fruits and/or vegetables per day. Based on their responses to the FFQ, participants reported a mean total fiber intake of 14.6 grams per 1000 kcal per day, which meets the Dietary Guidelines for Americans fiber recommendation of 14 g per 1000 kcal. This level of intake is 73.8% higher than that of average American adults.^58^ The mean insoluble fiber intake was 11.4 grams per 1000 kcal per day, and soluble fiber was 4 grams per 1000 kcal per day. Fruit and vegetable intake among participants showed a mean total fruit intake of 2.1 cup equivalents per day and a mean total vegetable intake of 3.6 cup equivalents per day, exceeding, on average, the 5-a-day recommendation to consume five servings of fruits and vegetables per day.

The participants’ adherence to the Healthy Eating Index (HEI) was assessed by calculating their average scores across various dietary components in the FFQ (Table S5†). Overall, the mean HEI score was 74.8, approximately 31% higher than the average score for this age group in the US.^59^ In terms of specific food groups, the HEI subscores (in average cup equivalents per 1000 kcal consumed) were 4.3 for fruits, 4.8 for vegetables, and 4.9 for greens and beans (65.4%, 41.2% and 44.1% higher, respectively, than average American intake). Whole grain consumption averaged 4.2, 82.6% higher than average Americans. In summary, the diet of study participants was of substantially higher quality than the average American with higher intake of the types of foods expected to provide complex substrates to gut bacteria.

None of the participants took either of the fiber supplements used in the fecal fermentations. Among the foods tested, several were consumed by the participants in the 24 hours before stool collection or were reported being consumed over the previous 12 months. All participants had consumed legumes in some form in the 24 hours before collecting stool and all reported consuming legumes regularly in the past year, ranging from 0.15 to 2.8 cup equivalents per day. Three of the participants consumed one banana or more in the 24 hours before stool collection and 4 reported eating them regularly, ranging from 2–3 times a month to daily in the past year. None of the participants reported eating flaxseed, coconut flour or kale before their stool collection, though other greens were consumed. The Block FFQ does not ask about kale or coconut flour and flaxseed is counted in a category with walnuts so we have no information about habitual kale, coconut flour or flaxseed intake among our participants.

### Even among participants with diverse fruit and vegetable diets, fecal microbiomes reveal variable microbial enzyme diversity

**Fig. 1:**
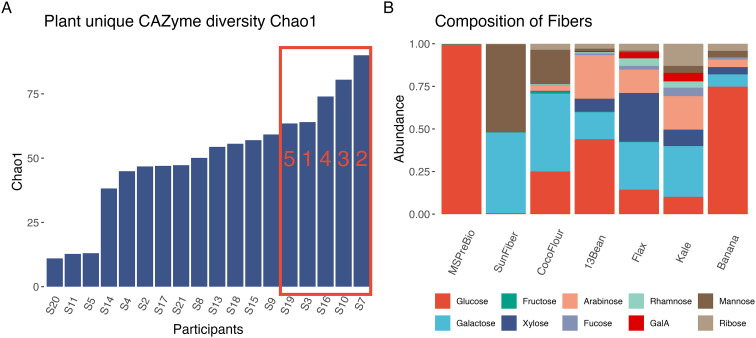
Characterization of microbiomes and dietary fibers for fermentations. (A) Chao1 diversity of plant unique CAZymes in the metagenomes of the 18 participants, with each bar representing one participant. The top 5 were chosen for the fecal fermentations with the fermentation participant IDs on the bar. (B) Monosaccharide composition of the 7 fermentation fibers.

After processing raw reads from the shotgun metagenomes of the 18 participants, the resulting metagenomes contained an average of 29.49 million reads per sample (sd = 0.88 million) (Table S10†). The metagenomes were mapped to the CAZy database and annotated by substrate. Because some CAZymes are ambiguous for plant or animal substrate, diversity metrics were computed using the plant-unique CAZymes (see Methods). Despite originating from participants with high-quality diets diverse in plants, the fecal microbiomes varied in plant-unique CAZyme diversity (Fig. 1A and Fig. S2, S3†). To determine the variability in fermentation of diverse fiber types, the stool samples from the five participants with the greatest capacity to degrade diverse carbohydrates—those with highest plant CAZyme Chao1 diversity—were selected as inoculants for fermentation experiments (samples 7, 10, 16, 3 and 19 in Fig. 1A). While any of the three calculated diversity metrics could have been used to identify the top 5 most diverse CAZyme repertoires, we chose the Chao1 metric because it is a richness estimator that is sensitive to rare species. Regardless of the metric chosen, the 5 selected for fecal fermentations were not “low diversity” by any of the metrics.

### Fecal fermentations with diverse fiber types show most change in microbial diversity and SCFA production occurs in the first 24 hours

Based on monosaccharide data from the Davis Food Glycopedia,^23^ we selected seven fibers with different monosaccharide profiles (Fig. 1B and Table S11†) for fecal fermentations. MS Prebiotic, a type 2 resistant starch made from potatoes, was almost entirely glucose. The fiber supplement Sunfiber is a guar gum galactomannan and was accordingly composed of galactose and mannose. Coconut flour was high in galactose, mannose, and glucose. Increasing in monosaccharide diversity, the 13 Bean soup was high in glucose, arabinose and galactose with some xylose and ribose as well. Flax was high in xylose, galactose, and glucose with some arabinose, rhamnose, galacturonic acid and ribose. Kale contained galactose, arabinose, xylose, and glucose, with a higher percentage of fucose and ribose than any other fiber. Finally, banana was mostly glucose with some galactose and still smaller amounts of xylose, arabinose, mannose, and ribose. These seven fibers were chosen as substrates in the fecal fermentation experiments which were conducted over a 72-hour period with sampling every 24 hours.

**Fig. 2:**
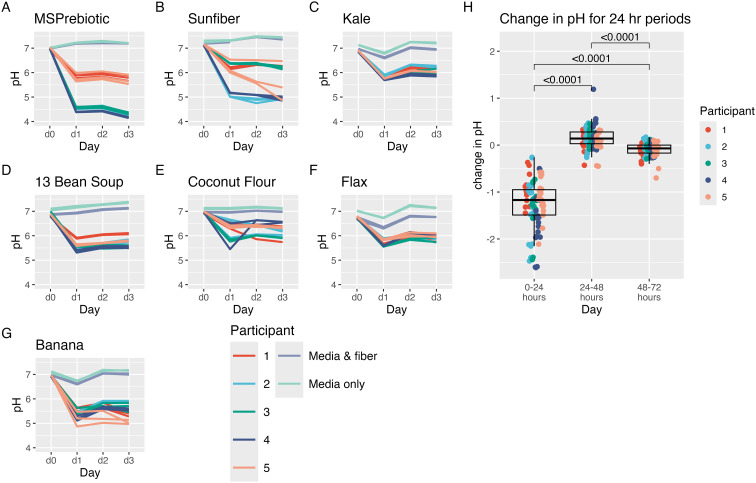
The change in pH over time in fecal fermentations of (A) MSPrebiotic, (B) Sunfiber, (C) kale, (D) 13 bean soup, (E) coconut flour. (F) flax, and (G) banana. (H) The change in pH for the sampling periods, Wilcoxon signed-rank tests with Bonferroni correction.

**Fig. 3:**
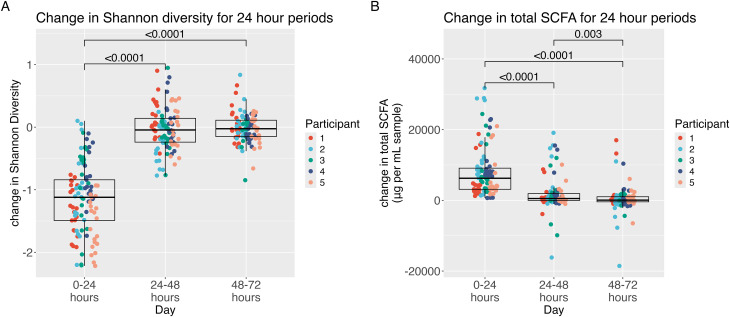
The change over the 24 hours time periods in fecal fermentations for (A) Shannon diversity and (B) total SCFA. Wilcoxon signed-rank tests with Bonferroni correction.

The pH of the fecal fermentations changed over the sampling time periods (Fig. 2A–G), decreasing the most in the first 24 hours (Fig. 2H). 16S sequencing from the fecal fermentations showed that the Shannon diversity of microbial taxa decreased the most in the first 24 hours of fermentation (Fig. 3A). The change in Shannon diversity over time is shown in Fig. S4.† Likewise, the greatest SCFA production (acetate, propionate, and butyrate) occurred in the first 24 hours of fermentation, with limited increases, if any, at later time points (Fig. 3B). The changes in total SCFAs over time for each substrate are shown in Fig. S5.†

**Fig. 4:**
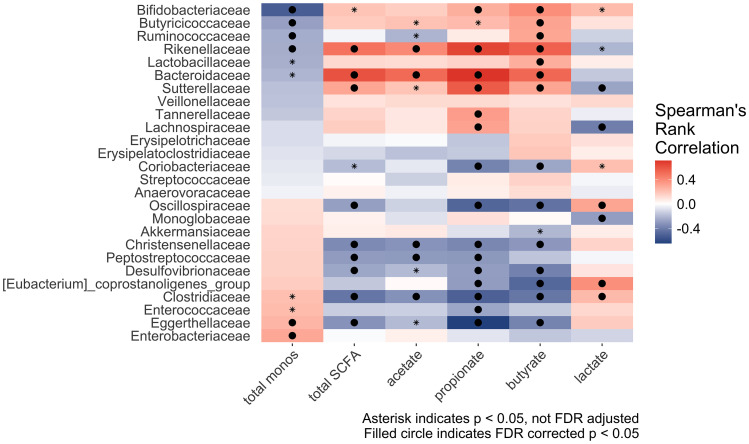
Correlation matrix between center log ratio transformed abundance of taxa present at 24 hours with minimum prevalence of 0.3 and different fermentation outcomes. Spearman's rank correlation with FDR adjustment.

Across all participants and fibers, bacterial abundance at 24 hours correlated with the amount of fermentation (change in total monosaccharides and change in SCFAs and lactate) from *Bifidobacteriaceae*, *Butyricicoccaceae*, and *Ruminococcaceae* with the highest fermentation down to *Clostridiaceae*, *Enterococcaceae*, and *Eggerthellaceae* with the lowest fermentation (Fig. 4).

### Changes in microbial diversity, free monosaccharides, and SCFA production are variable by fiber type

**Fig. 5:**
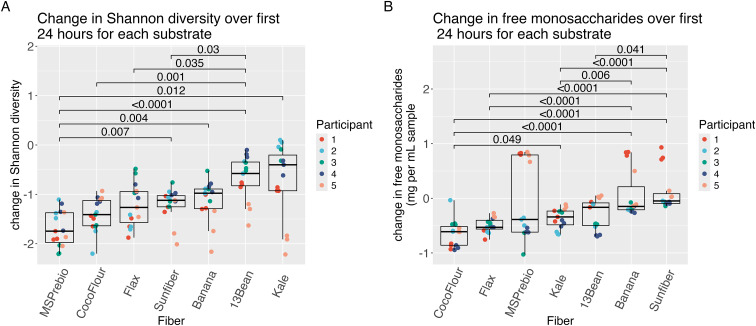
(A) The change in Shannon diversity in the first 24 hours for each substrate. (B) The change in free monosaccharides in the first 24 hours for each substrate. Wilcoxon signed-rank tests with Bonferroni correction.

Given that the greatest changes occurred in the first 24 hours, we next compared the differences among substrates during this time period. Fermentation with the resistant starch supplement MS Prebiotic resulted in a significantly larger decrease in Shannon diversity in the first 24 hours than kale, 13-bean soup, banana, or the galactomannan supplement, Sunfiber (Fig. 5A). Coconut flour, flax, and Sunfiber likewise elicited a significantly greater decrease in microbial diversity than 13-bean soup. Similar findings were observed when measuring Pielou's evenness (Fig. S6†).

As another indicator of the fermentation process, total (bound plus unbound) and free (unbound) monosaccharides were measured. For changes in free monosaccharides (Fig. 5B), coconut flour had a significantly larger decrease than kale, banana and Sunfiber. Banana had a significantly larger decrease in total monosaccharides than flax, kale, and coconut flour (Fig. S7†).

**Fig. 6:**
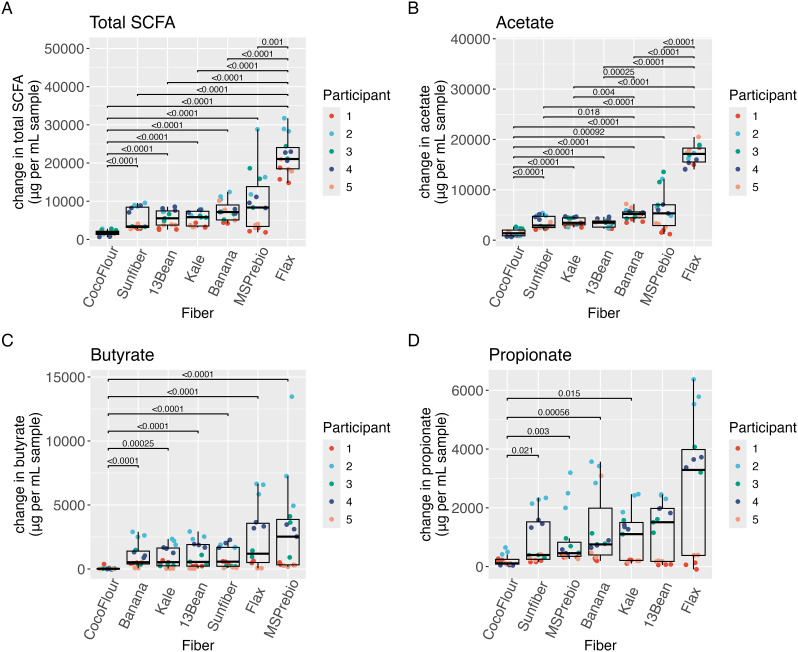
Changes by fiber in first 24 hours of (A) total short chain fatty acids, (B) acetate, (C) butyrate, (D) propionate. Wilcoxon signed-rank tests with Bonferroni correction.

SCFA production differed by substrate (Fig. 6). Coconut flour yielded the lowest production of the three main SCFAs (acetate, propionate and butyrate) in the first 24 hours of fermentation, while flax yielded the highest (Fig. 6A). For flax, this was driven by large production of acetate (Fig. 6B). Coconut flour had the lowest production of butyrate (Fig. 6C) and lower propionate production than kale, banana, MS Prebiotic and Sunfiber (Fig. 6D). Most fermentations had very small increases in lactate in the first 24 hours (Fig. S8†) with MS Prebiotic resulting in significantly more lactate than every fiber but flax. The concentration of lactate over time is shown in Fig. S9.†

**Fig. 7:**
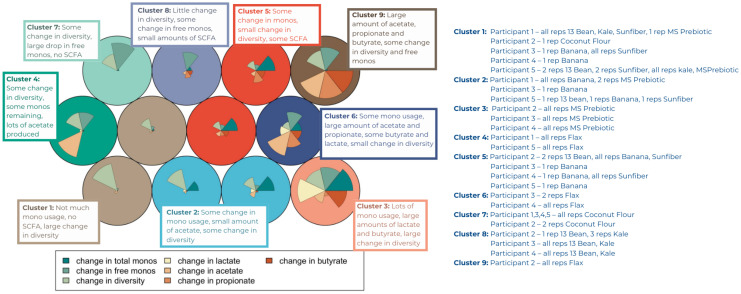
Nine cluster Self Organizing Map (SOM) using the 24-hour change in total monosaccharides, propionate, acetate, butyrate, free monosaccharides, lactate, and Shannon diversity and the sample composition of the clusters.

A self-organizing map (SOM) was used to group samples based on their similarity using the fermentation outcomes of the 24-hour change in total monosaccharides, acetate, propionate, butyrate, lactate, free monosaccharides, and Shannon diversity (Fig. 7). The number of clusters (9) for the SOM was selected based on the lowest within cluster sum of squares (Fig. S10†). These 9 clusters demonstrate that the 35 fermentation experiments had fiber-specific and donor-specific effects on the extent of fermentation in the first 24 hours ranging from limited primary degradation (liberated monosaccharides) to little change in microbial diversity to robust production of SCFAs.

### Fermentation with diverse fiber types show that changes in microbial diversity and SCFA production vary by donor, despite donor selection for high CAZyme diversity

**Fig. 8:**
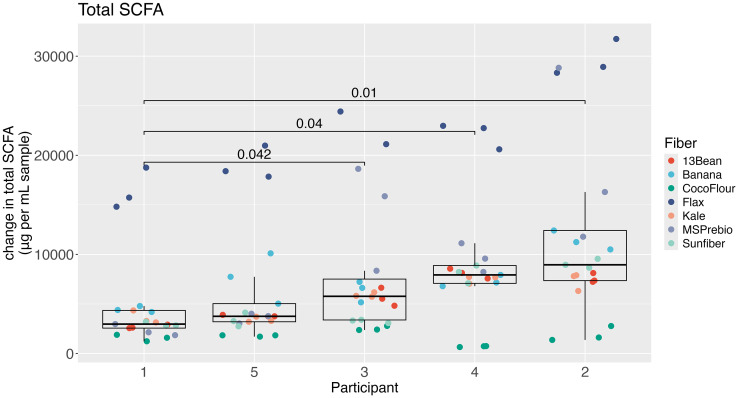
Changes by participant in first 24 hours of total short chain fatty acids. Wilcoxon signed-rank tests with Bonferroni correction.

Donor-specific fermentations differed by changes in microbial diversity (Fig. 5A) but were more apparent by changes in total SCFAs (Fig. 8). Participant 1 produced significantly less total SCFAs than participants 2,3 and 4. For acetate, participant 1 also produced significantly less than participant 3 (Fig. S11A†). There were more differences seen in butyrate production (Fig. S11B†) with participant 5 producing significantly less than all other participants and participant 2 producing significantly more than participants 1, 3 and 5. For propionate, participant 1 and participant 5 produced significantly less than participants 2, 3 and 4, with participant 2 producing significantly more propionate than all other participants (Fig. S11C†). Participant 2 produced significantly less lactate than participant 1, 3 and 5 (Fig. S11D†). Despite the differences in production of the individual short chain fatty acids, a pattern of responsiveness becomes visible, with participants 2, 3 and 4 more responsive than participants 1 and 5.

Differences in starting metagenome microbial composition between the responders (participants 2, 3, and 4) and the non-responders (participants 1 and 5) were tested with permutational analysis of variance (or PERMANOVA) of Bray–Curtis distances using the R package vegan. The response group was not significantly associated with the dissimilarity of the metagenome composition (*p* = 0.4). Because individual taxa matter less than functions when it comes to degrading carbohydrates we also tested for differences between responders and non-responders in glycoside hydrolase and polysaccharide lyase CAZyme family abundance. Here again, there was not a significant association with response group (*p* = 0.4).

## Discussion

To study the degradation of complex food fibers and supplement fibers, we recruited healthy adults who regularly eat fruits and vegetables and selected the five participant microbiomes with the highest plant CAZyme diversity for *in vitro* fermentation experiments. In our fecal fermentations, the largest changes in pH, microbial diversity and SCFA production occurred in the first 24-hour period. Fermentation outcomes varied by fiber type with larger than expected differences by donor.

As expected, SCFA production varied by substrate. Flaxseed resulted in the highest SCFA concentration, and this was correlated with increased production of acetate. A previous study of flaxseed fermentation with pooled inocula from three donors found that mainly acetate was produced.^60^ Another *in vitro* study of flaxseed fermentation also found that acetate was the predominant SCFA.^61^ Coconut flour was not well fermented by any of the participants in our study, producing the lowest amount of SCFAs. Coconut flour is not widely consumed in the US, except by those on gluten-free or ketogenic diets. Other studies have found poor fermentation with less commonly consumed fibers. Carrageenan, furcellaran and psyllium are not common in the North-European diet and weren't fermented by the pooled microbiota of the 7 local participants.^62^ “Unconventional” fiber sources like rice hulls, bamboo and algae were not fermented well in a study of 22 fiber sources fermented with the microbiotas of 3 healthy stool donors.^63^

While monosaccharide composition is not a complete characterization of fiber structure it is a useful first step in understanding the differences in fiber types and their fermentation outcomes. Variation in fermentation by monosaccharide composition of the fiber is consistent with the few studies that have measured monosaccharides. Parkar *et al.*, found that the monosaccharide composition of foods drove the metabolic and microbiome profile *in vitro*, with glucose availability as the primary modulator of the microbiome.^64^ Another study found that maize particle size fractions with more glucose, mannose and galactose fermented better, with more gas and SCFA production and a larger drop in pH.^65^ Monosaccharide content predicted pH, microbial composition and diversity in study of 55 fibers *in vitro* fermented with pooled feline fecal inocula.^45^ Glucose and xylose, in particular, were associated with the reduction in pH. Fibers with a similar monosaccharide composition were found to have similar fermentation outcomes. But intriguingly, the study found the monosaccharide composition (and presumably, the carbohydrate structure) could vary considerably between similar foods, such as two types of sweet potatoes. And very different foods, such as radish and buckwheat, could have similar monosaccharide compositions. These surprising observations highlight the importance of this kind of food profiling.

The fiber, fruit and vegetable intake and overall diet quality of our participants likely played a role in their microbiotas’ ability to degrade the test fibers. Many human studies have shown that the background or habitual diet of participants in fiber interventions can affect the outcome.^66–68^ This effect is also seen in fecal fermentations. Brahma *et al.*, performed *in vitro* fermentations of grain fibers with stool from people with different diet quality. The higher diet quality group had more diversity and beneficial microbes and could better ferment the grain. Both groups’ microbiotas could produce SCFAs but the higher diet quality group made more butyrate and lower quality group made more acetate and propionate.^69^ For these reasons, some have called for baseline microbiota and habitual diet to be taken into consideration in fiber studies^70^

The effect of fiber consumption on the gut microbiome in humans is highly variable. Wastyk *et al.*, observed an increase in CAZyme abundance when participants increased fiber intake by 20 grams per day over 10 weeks^71^ however SCFA levels did not change. In another study, a two-week fiber intervention that doubled participants’ fiber intake resulted in no change in individual CAZymes, CAZyme diversity or abundance, and no change in fecal SCFA levels.^72^ An interventional study of snack bars containing 1, 2 or 4 different fibers (30 g of fiber total daily for 3 weeks) reported increases in CAZyme abundance.^73^ The same group's study of pea and orange fiber snack bars also revealed increases in CAZyme abundance but no change in SCFAs.^74^ However, such studies generally don't recruit for a particular baseline diet and it's difficult to determine mechanisms without companion *in vitro* fermentations.

One's habitual diet may play a role in the variable effect of fiber as well, “priming” the microbiota to respond to fiber or creating a resilient community resistant to change when nutrients are increased or altered. A study of inulin supplementation over 3 weeks in high and low fiber consumers found greater microbial “responsiveness” in the habitual high fiber consuming participants, as measured by changes in microbial composition, though there was no significant increase in SCFAs.^68^ However, a study by Holmes *et al.* found that participants who habitually ate more fiber also made proportionally less butyrate when fed three monomerically different prebiotics, a result the researchers suggest implies that there are fixed caps in other nutrients involved in carbohydrate fermentation.^75^ Additionally, carbohydrate structures that are less common in the diet, such as xyloglucans isolated from cell walls, may create larger changes in the composition of the gut microbiota than more commonly consumed compounds like pectin.^76^ We attempted to control for this by recruiting participants who regularly consumed 5 fruits and vegetables a day and selecting the 5 with the most diverse plant CAZymes in their microbiomes but still found differences in fiber fermentation capacity.

In the current study, all donors met the DGA guidelines for average fiber intake as well as servings of fruits and vegetables. Additionally, HEI scores of their baseline diets showed higher diet quality than the average American's. We used only the fecal inoculum from those five participants with the highest plant CAZyme diversity. Despite this stringent donor criteria, fermentations differed considerably by donor. The self-organizing map illustrated that samples clustered commonly by participant, with participants 1 and 5 together, and participants 2,3 and 4 together. On most fibers, participants 2, 3, and 4 were able to use more of the carbohydrates and produce more SCFAs as compared to participants 1 and 5. This was especially visible with the fiber supplements, Sunfiber and MSPrebiotic, in the pH profiles in Fig. 2A–H. Using the participants’ initial stool sample metagenomes, we ran a PERMANOVA both on taxa and CAZyme families. Neither was significant, but this is most likely due to the small sample size (*n* = 5) which does not have enough power to distinguish differences. However, a recent study found that in *Bifidobacterium*, genes encoding substrate binding proteins and permeases were more predictive of responder phenotypes than CAZymes.^77^

In a cross-sectional study of healthy adults, increased diversity of non-glucose monosaccharides in the diet was associated with lower GI inflammation.^78^ Further, specific dietary monosaccharides were associated with increases in specific microbial taxa, implying that perhaps dietary changes could tailor the microbiota. In the current study, some fermentations were less productive of SCFAs, even with donors consuming at least five fruits and vegetables per day, implying that diet alone might not be enough. Indeed, in healthy adults, the fecal microbiome was a much stronger predictor of fecal SCFAs than any aspect of dietary intake.^79^ Another study found that fiber intake correlated with carbohydrates found in the participants’ stool, possibly indicating that the participants may have lacked the necessary enzymes to ferment the fiber.^71^ This poses the question of whether we need to push our dietary intake of diverse fibers further than “5 a Day” or the DGA recommendation of 14 g per 1000 kcal towards the levels consumed by hunter-gatherer cultures.

Does every human gut contain the taxa, or more importantly, the functions necessary to degrade common dietary fibers? Droplet tests of inulin, GOS, dextrin and xylan found that the microbiomes of 9 participants were capable of degrading them with the primary degraders found in most individuals.^80^ However, there were up to 25-fold variations in composition and abundance of monosaccharide-consuming taxa. This may explain why some of the fermentations in the current study seemed to stall after primary degradation (*e.g.* clusters in the self-organizing map with high monosaccharides and lower SCFAs).

Across all samples, a higher abundance of *Bifidobacteriaceae*, *Butyricicoccaceae*, and *Ruminococcaceae* was associated with the highest fermentation, a greater decrease in total monosaccharides and greater production of SCFA. Members of *Bifidobacterium* and *Ruminococcus* are well known to be primary degraders of resistant starch^81^ and these families were increased in participants who were able to degrade MSPrebiotic (data not shown). This finding suggests that taxa such as *Bifidobacterium* and *Ruminococcus* may be important targets for improving the degradation of resistant starch and increasing SCFA production in adult gut microbiomes.

Our study has several strengths. We investigated monosaccharide-diverse whole foods as well as two prebiotic fiber supplements. Our *in vitro* fermentations allowed for the precise measurement of SCFA concentrations. With advanced, high-throughput carbohydrate analysis, we were able to characterize not only the monosaccharide composition of our digested and dialyzed fibers but also track the monosaccharide concentrations over time in our fermentations. However, *in vitro* batch fermentations cannot perfectly replicate the environment of the human gut. Unlike in the colonic environment, with continuous resupply of nutrients and host absorption of SCFAs, the conditions *in vitro* can restrict microbial growth, changing the composition of the microbiota and affecting its metabolism. We limited most of our analysis to the first 24 hours of fermentation to attempt to account for this.

## Conclusions

Even frequent consumers of fruits and vegetables with diverse CAZyme repertoires demonstrate variable capacity for fiber fermentation, depending on the fiber and the presence of specific bacterial taxa. Our data show that there is considerable variation in the extent to which gut microbiota from consumers of fruits and vegetables are able to ferment diverse fibers, with some producing much more SCFAs than others. These data suggest a precision nutrition or probiotic approach, focusing on specific fiber-containing whole foods and targeting primary degraders to ensure that gut microbiomes can utilize fiber's benefits.

## Author contributions

Conceptualization: D. A. M., D. G. L., C. L. B. and J. T. S.; methodology, investigation: K. M. K., C-Y. W., C. S., I. R. S., Y. T., S. C., S. J., K. C. and K. D-M.; formal analysis: S. E. B.; writing – original draft: S. E. B., D. A. M. and D. G. L.; supervision: D. A. M., D. G. L., C. L. B. and J. T. S.; funding acquisition: D. A. M., D. G. L., C. L. B. and J. T. S.; writing – reviewing and editing: J. T. S., K. M. K., C-Y. W., C. S., I. R. S., Y. T., K. C. and K. D-M. All authors reviewed and approved the final manuscript.

## Conflicts of interest

D.A.M. and C.B.L. are cofounders of Infinant Health, a probiotic company, and One.Bio, a company advancing novel bioactive glycans. Neither of these companies had any role in the conceptualization, design, analysis, or preparation of the manuscript.

## Supplementary Material

FO-016-D5FO00947B-s001

## Data Availability

SCFAs, monosaccharides and pH data are deposited in https://github.com/sblecksmith/GENIUS_project/tree/clean/data. Metagenomic reads for the 18 participants in the GENIUS study are deposited in the NCBI Sequence Read Archive (SRA) under the BioProject accession number PRJNA816918 (https://www.ncbi.nlm.nih.gov/sra/PRJNA816918). Reads from the 16S sequencing of fermentation samples are available under BioProject accession number PRJNA1182822 (https://www.ncbi.nlm.nih.gov/sra/PRJNA1182822) Scripts used in this project to conduct the analyses and generate the figures are available at https://github.com/sblecksmith/genius_project.
