# Supplementary material for: Fecal microbiomes from healthy adult consumers of fruits and vegetables exhibit fiber- and donor-specific fermentation: “5 a day” is not enough
Source: Food Funct. 2025 Sep 8;16(19):7561–77. doi: 10.1039/d5fo00947b (PMC12415832; doi:10.1039/d5fo00947b)
Supplement: FO-016-D5FO00947B-s001 [file FO-016-D5FO00947B-s001.pdf]

Supplementary Information (SI) for Food & Function.
This journal is © The Royal Society of Chemistry 2025

Supplemental Tables and Figures

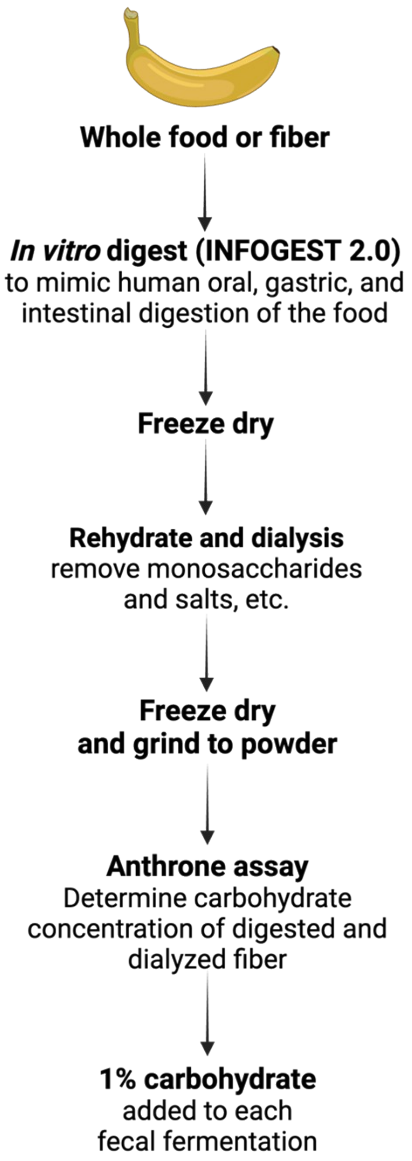

Figure S1. Fiber digestion and processing for fecal fermentations.

Table S1. Participant demographics and characteristics

	Mean	SD	Min	Max
Age (years)	30.64286	7.947976	20	47
Sex, % (n)				
Female	61.1	11		
Male	38.9	7		
Ethnicity %, (n)				
Hispanic or Latino	16.7	3		
Not Hispanic or Latino	83.3	15		
Race %, (n)				
White	61.1	11		
Asian	22.2	4		
Unsure	5.6	1		
Other	11.1	2		
Education %, (n)				
Some college	22.2	4		
Bachelor's degree	50.0	9		
Master's degree	11.1	2		
Professional or doctorate degree	16.7	3		
Marital status %, (n)				
Single/never married	50.0	9		
Married/Unmarried couple	33.3	6		
Divorced/Separated	11.1	2		
Refuse	5.6	1		
Born in the U.S. %, (n)				
Yes	72.2	13		
No	27.8	5		
Country of Birth %, (n)				
Canada	5.6	1		
Brazil	5.6	1		
Moldova	5.6	1		
Vietnam	11.1	2		
Country of living the most %, (n)				
U.S.	83.3	15		
Canada	5.6	1		
Brazil	5.6	1		
Vietnam	5.6	1		
Years living in the U.S.	27.7	11.7	8.0	50.0

Table S2. Participant reported general health history

	Mean	SD	Min	Max
Current weight (lb)	152.8	31.7	104	218
Breastfed %, (n)				
Yes	83.3	15		
No	11.1	2		
Unsure	5.6	1		
Age when stopped consuming breastmilk %, (n)				
Between 1-3 months old	5.6	1		
Between 3-6 months old	16.7	3		
Between 6-12 months old	11.1	2		
More than 12 months old	11.1	2		
Unsure	38.9	7		
Birth delivery method %, (n)				
Vaginally, not delivered during a water birth	66.7	12		
C-section, elective	22.2	4		
Unsure	11.1	2		
Courses of oral antibiotics in the past 10 years %, (n)				
0	27.8	5		
1 to 3	55.6	10		
4 to 6	11.1	2		
7 to 10	5.6	1		
Probiotic supplements intake in the last year %, (n)				
Yes	16.7	3		
No	77.8	14		
Unsure	5.6	1		

Table S3. Participant reported diet intake history

	%	n
Intake alcohol frequency		
5 to 6 days per week	5.6	1
3 to 4 days per week	16.7	3
2 days per week	11.1	2
1 day per week	16.7	3
2 to 3 days per month	16.7	3
1 day per month	11.1	2
I did not drink any alcohol in the past month	22.2	4
Alcoholic drinks on a typical day		
0 drink	22.2	4
1 drink	50.0	9
2 drinks	22.2	4
3 to 4 drinks	5.6	1
Intake red wine frequency		
1 day per week	38.9	7
2 to 3 days per month	22.2	4
I did not drink any red wine in the past mont	16.7	3
Glasses of red wine on a typical day		
0 glasses	38.9	7
1 glass	33.3	6
2 glasses	27.8	5
Intake coffee frequency*		
1 to 2 cups per day	61.1	11
3 to 4 cups per day	5.6	1
1 to 2 cups per week	5.6	1
3 to 4 cups per week	11.1	2
2 to 3 cups per month	5.6	1
I did not drink any coffee in the past month	11.1	2
Intake black or green tea frequency		
1 to 2 cups per day	22.2	4
3 to 4 cups per day	11.1	2
1 to 2 cups per week	27.8	5
3 to 4 cups per week	11.1	2
5 to 6 cups per week	5.6	1
2 to 3 cups per month	11.1	2
1 cup per month	5.6	1
I did not drink any tea in the past month	5.6	1
Intake dark chocolate fequency		
Daily	5.6	1
5 to 6 times per week	11.1	2
3 to 4 times per week	5.6	1
2 times per week	27.8	5
1 time per week	5.6	1
2 to 3 times per month	16.7	3
1 time per month	5.6	1
I did not eat any dark chocolate in the past month	22.2	4

Table S4. Participant reported frequency for the consumption of foods and beverages containing microbes

		%	n
Belgian lambics			
	Seldom	77.8	14
	Never	11.1	2
	Unsure	11.1	2
Fermented pickled foods			
	2 days per week	16.7	3
	Seldom	50.0	9
	A few days per month	11.1	2
	Never	16.7	3
	Unsure	5.6	1
Fruit juices or sodas with probiotics			
	Seldom	27.8	5
	Never	72.2	13
Kefir			
	3-4 days per week	5.6	1
	A few days per month	5.6	1
	Seldom	22.2	4
	Never	66.7	12
Kimchi			
	A few days per month	22.2	4
	Seldom	50.0	9
	Never	27.8	5
Kombucha			
	2 days per week	5.6	1
	A few days per month	16.7	3
	Seldom	50.0	9
	Never	27.8	5
Miso			
	2 days per week	5.6	1
	A few days per month	22.2	4
	Seldom	55.6	10
	Never	16.7	3
Mixed powders with probiotics			
	3-4 days per week	5.6	1
	Seldom	11.1	2
	Never	83.3	15
Sauerkraut			
	1 day per week	5.6	1
	A few days per month	5.6	1
	Seldom	16.7	3
	Never	72.2	13
Tempeh			
	1 day per week	5.6	1
	A few days per month	11.1	2
	Seldom	5.6	1
	Never	72.2	13
Unfiltered beer			
	1 day per week	16.7	3
	3-4 days per week	5.6	1
	A few days per month	5.6	1
	Seldom	22.2	4
	Never	50.0	9
Traditional yogurt			
	1 day per week	11.1	2
	2 days per week	5.6	1
	3-4 days per week	11.1	2
	A few days per month	22.2	4
	Seldom	38.9	7
	Never	11.1	2
Yogurt with added probiotics			
	3-4 days per week	5.6	1
	5-6 days per week	5.6	1
	Seldom	33.3	6
	Never	55.6	10

Table S5. Participant HEI scores and subscores from FFQ

HEI	Mean	SD
Total	74.8	6.6
Fruits (cup eq./1000 kcal)	4.3	1.0
Vegetables (cup eq./1000 kcal)	4.8	0.4
Legumes (cup eq./1000 kcal)	4.9	0.4
Protein (cup eq./1000 kcal)	4.8	0.3
Whole grains (cup eq./1000 kcal)	4.2	2.5
Refined grains (cup eq./1000 kcal)	9.2	1.3
Dairy (cup eq./1000 kcal)	6.2	2.7
Fatty acids (g/1000 kcal)	7.4	3.1
Sodium (g/1000 kcal)	3.1	2.7
Added sugar (g/1000 kcal)	9.4	1.2
Saturated fat (g/1000 kcal)	6.8	2.6

Table S6. Participant energy and standard nutrients from FFQ

	Mean	SD	Min	Max
Food energy (kcal)	2100.0	1077.5	1141.8	5229.9
Protein (g)	79.2	40.1	36.9	187.0
Carbohydrates (g)	254.5	141.5	151.7	721.6
Fat (g)	87.5	43.6	38.6	189.2
Cholesterol (g)	247.3	151.7	91.5	528.4
Dietary fiber (g)	31.6	22.5	16.3	96.0
Sugars (g)	105.7	39.1	62.1	210.4
Alcohol (g)	6.0	7.2	0.0	26.6

Table S7. Participant vitamins and mineral intake from FFQ

	Mean	SD	Min	Max
Vitamins				
Vitamin A (mcg RAE)	1251.9	766.9	415.2	3186.4
Retinol (mcg)	495.3	298.7	181.5	1166.3
Alpha-carotene (mcg)	996.7	731.5	261.9	2684.1
Beta-carotene (mcg)	8465.9	7370.0	2413.0	29551.7
Cryptoxanthin, beta (mcg)	230.8	127.4	47.3	534.3
Lycopene (mcg)	6275.6	4610.6	1629.6	19132.4
Lutein/Zeaxanthin (mcg)	7587.1	8357.2	1460.7	37369.6
Vitamin E as a alpha-tocopherol (mcg)	12.7	7.1	6.2	30.1
Vitamin E added (mcg)	0.7	1.0	0.0	2.9
Vitamin D (D2 + D3) (mcg)	5.6	3.6	1.6	15.5
Vitamin K as phylloquinone (mcg)	430.5	425.3	103.5	1927.3
Vitamin C (mg)	166.5	83.3	36.6	344.1
Thiamin (vitamin B1) (mg)	1.8	1.1	0.9	5.2
Riboflavin (vitamin B2) (mg)	2.3	1.0	0.9	4.6
Niacin (mg)	22.5	12.9	11.0	58.4
Vitamin B6 (mg)	2.4	1.4	1.3	6.8
Total folate (mcg)	574.7	376.6	268.1	1616.5
Folate DFE (mcg)	661.2	442.0	313.1	1958.7
Folic acid (mcg)	124.3	110.2	36.1	491.6
Food folate (mcg)	450.4	292.2	203.4	1243.2
Vitamin B12 (mcg)	5.1	2.6	2.4	11.0
Vitamin B12 added (mcg)	1.2	1.3	0.1	4.6
Total choline (mg)	380.0	163.4	172.1	764.8
Minerals				
Calcium (mg)	1170.5	526.5	422.6	2123.9
Iron (mg)	16.7	10.5	7.4	48.2
Magnesium (mg)	439.3	254.5	215.9	1110.6
Phosphorus (mg)	1496.3	752.4	636.7	3527.8
Potassium (mg)	3851.4	1878.3	2188.9	9059.1
Sodium (mg)	3768.3	2255.2	1682.6	10535.1
Zinc (mg)	13.0	7.1	6.5	35.2
Cooper (mg)	2.1	1.1	1.0	5.2
Selenium (mg)	99.9	47.6	44.8	221.0

Table S8. Participant flavonoids and other compounds from FFQ

	Mean	SD	Min	Max
Total flavonoids (mg)	374.6	300.9	124.6	1244.0
Total anthocyanidins (mg)	39.9	22.5	7.3	98.2
Cyanidin (mg)	8.5	5.7	1.2	21.1
Delphinidin (mg)	8.0	4.2	1.4	17.9
Malvidin (mg)	13.4	9.8	1.7	37.6
Pelargonidin (mg)	1.6	0.8	0.6	3.1
Peonidin (mg)	6.8	10.4	1.2	39.2
Petunidin (mg)	1.7	0.9	0.3	3.3
Total flavan-3-ols (mg)	261.3	257.4	21.1	1003.6
Epicatechin (mg)	15.0	9.6	4.6	41.3
Epicatechin 3-gallate (mg)	15.9	17.1	0.6	65.3
Epigallocatechin (mg)	24.6	26.7	1.0	102.4
Epigallocatechin 3-gallate (mg)	45.9	55.4	1.1	209.5
Catechin (mg)	13.6	7.1	4.2	32.5
Gallocatechin (mg)	2.2	2.1	0.1	7.7
Theaflavin (mg)	2.0	2.1	0.0	6.5
Theaflavin-3,3'-digallate (mg)	2.2	2.3	0.0	7.2
Theaflavin-3'-gallate (mg)	1.8	1.9	0.0	6.2
Theaflavin-3-gallate (mg)	1.6	1.7	0.0	5.1
Thearubigins (mg)	136.4	139.7	1.2	521.8
Total flavanones (mg)	19.8	22.3	1.7	96.7
Eriodictyol (mg)	0.1	0.0	0.0	0.2
Hesperetin (mg)	11.0	12.3	1.0	52.7
Naringenin (mg)	8.8	10.2	0.8	43.9
Total flavones (mg)	2.6	1.6	0.7	5.8
Apigenin (mg)	0.5	0.4	0.1	1.5
Luteolin (mg)	2.1	1.3	0.5	4.4
Total flavonols (mg)	38.6	27.6	13.8	115.7
Isorhamnetin (mg)	2.5	2.0	0.6	7.9
Kaempferol (mg)	9.9	8.4	1.6	32.8
Myricetin (mg)	2.8	2.7	0.5	11.4
Quercetin (mg)	23.4	15.6	8.9	69.3
Total isoflavones (mg)	12.2	14.2	0.2	54.2
Daidzein (mg)	5.0	5.9	0.1	23.1
Genistein (mg)	6.5	7.5	0.1	28.7
Glycitein (mg)	0.8	0.8	0.0	2.5
Others (mg)				
Caffeine (mg)	188.9	128.7	4.8	446.8
Theobromine (mg)	19.2	18.0	5.1	77.5

Table S9. Participant fatty acids intake from FFQ

	Mean	SD	Min	Max
Total saturated fatty acids (g)	25.5	15.0	10.3	63.6
Butanoic, butyric 4:0	0.58	0.50	0.10	1.88
Hexanoic, caproic 6:0	0.34	0.31	0.05	1.22
Octanoic, caprylic 8:0	0.30	0.26	0.05	0.96
Decanoic, capric 10:0	0.49	0.41	0.09	1.51
Dodecanoic, lauric 12:0	0.89	0.77	0.19	2.97
Tetradecanoic, myristic 14:0	2.17	1.72	0.48	6.27
Hexadecanoic, palmitic 16:0	13.93	7.52	5.97	32.83
Octadecanoic, stearic 18:0	5.95	3.43	2.55	14.87
Total mono-unstaurated fatty acids (g)	32.2	15.7	13.0	66.0
Hexadecenoic, palmitoleic 16:1	1.01	0.54	0.48	2.36
Octadecenoic,oleic 18:1	30.51	14.91	12.17	62.18
Eicosenoic, gadoleic 20:1	0.29	0.15	0.14	0.65
Docosenoic, erucic 22:1	0.05	0.03	0.01	0.12
Total poly-unsaturated fatty acids (g)	22.7	12.4	11.1	45.1
Octadecadienoic, linoleic 18:2	19.53	10.60	9.51	39.89
Octadecatrienoic, linolenic 18:3	2.72	2.10	1.22	9.35
Octadecatetraenoic, parinaric 18:4	0.02	0.01	0.01	0.05
Eicosatetraenoic 20:4	0.10	0.07	0.03	0.25
Eicosapentaenoic, timnodonic 20:5 (EPA)	0.05	0.04	0.01	0.20
Docosapentaenoic, clupanodonic 22:5	0.02	0.01	0.00	0.07
Docosahexenoic 22:6 (DHA)	0.10	0.08	0.02	0.35

Table S10. The number of sequencing reads per sample and average read length before and after the pre-processing stages of the bioinformatics pipeline

	Average read length	Millions of sequencing reads per sample			
		Max	Min	Mean	Median
Raw read	151.00 (0.00)	54.25	33.61	40.27 (0.90)	39.02
BMTagger remove human DNA	151.00 (0.00)	54.13	33.58	40.20 (0.90)	38.98
Trimmomatic	150.70 (0.000078)	52.55	32.65	39.04 (0.87)	37.79
FastUniq remove duplicate reads	150.67 (0.000092)	41.42	26.82	31.62 (0.65)	30.92
FLASH merge reads	248.96 (0.0011)	38.83	24.82	29.49 (0.88)	28.77

Table S11. Monosaccharide composition of the selected fibers and foods

	Glucose	Galactose	Fructose	Xylose	Arabinose	Fucose	Rhamnose	GlcA	GalA	GlcNAc	GalNAc	Mannose	Allose	Ribose
MSPreBio	16.81046	0.00000	0.04805	0.00000	0.00000	0.00381	0.00000	0.00000	0.00000	0.00000	0.00000	0.00403	0.00000	0.02453
SunFiber	0.04629	3.64298	0.00685	0.00000	0.00000	0.00000	0.00083	0.00000	0.00000	0.00000	0.00000	3.98006	0.00000	0.02139
CocoFlour	0.44510	0.81002	0.02564	0.00356	0.05128	0.00444	0.01319	0.00000	0.00000	0.00000	0.00000	0.35826	0.00000	0.06317
13Bean	3.05670	1.09487	0.02867	0.52479	1.77461	0.07285	0.05266	0.01079	0.03712	0.00766	0.00052	0.10252	0.00000	0.19996
Flax	1.29930	2.51999	0.03336	2.57052	1.24170	0.19503	0.40189	0.00000	0.32321	0.00989	0.00000	0.08781	0.00000	0.36442
Kale	0.25188	0.73615	0.00000	0.23855	0.48623	0.12228	0.09139	0.01018	0.12423	0.00502	0.00000	0.10111	0.00000	0.32097
Banana	3.99347	0.38377	0.00000	0.22680	0.23808	0.04641	0.01487	0.00573	0.00574	0.01612	0.00124	0.20455	0.00000	0.22076

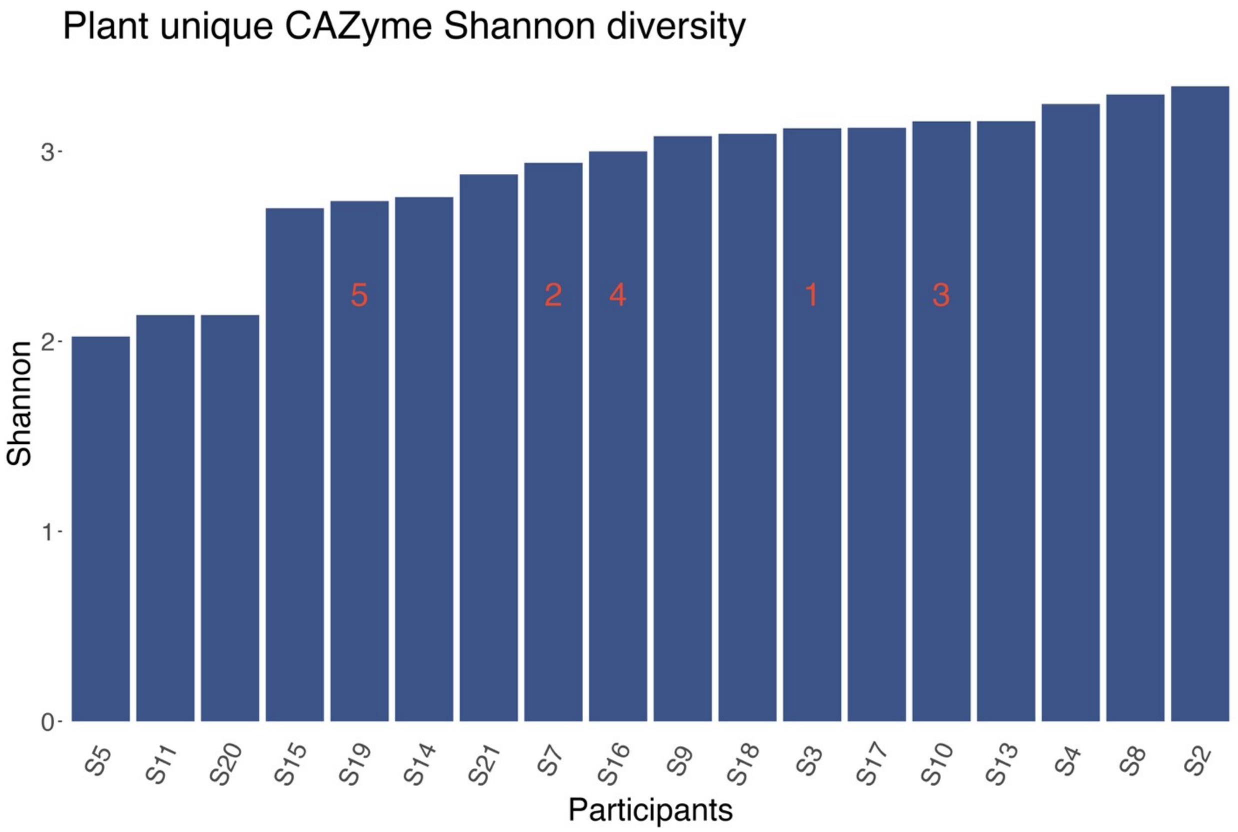

Figure S2. Shannon diversity of plant unique CAZymes in the metagenomes of the 18 participants, with each bar representing one participant. The 5 chosen for the fecal fermentations are labeled with the fermentation participant IDs on the bar.

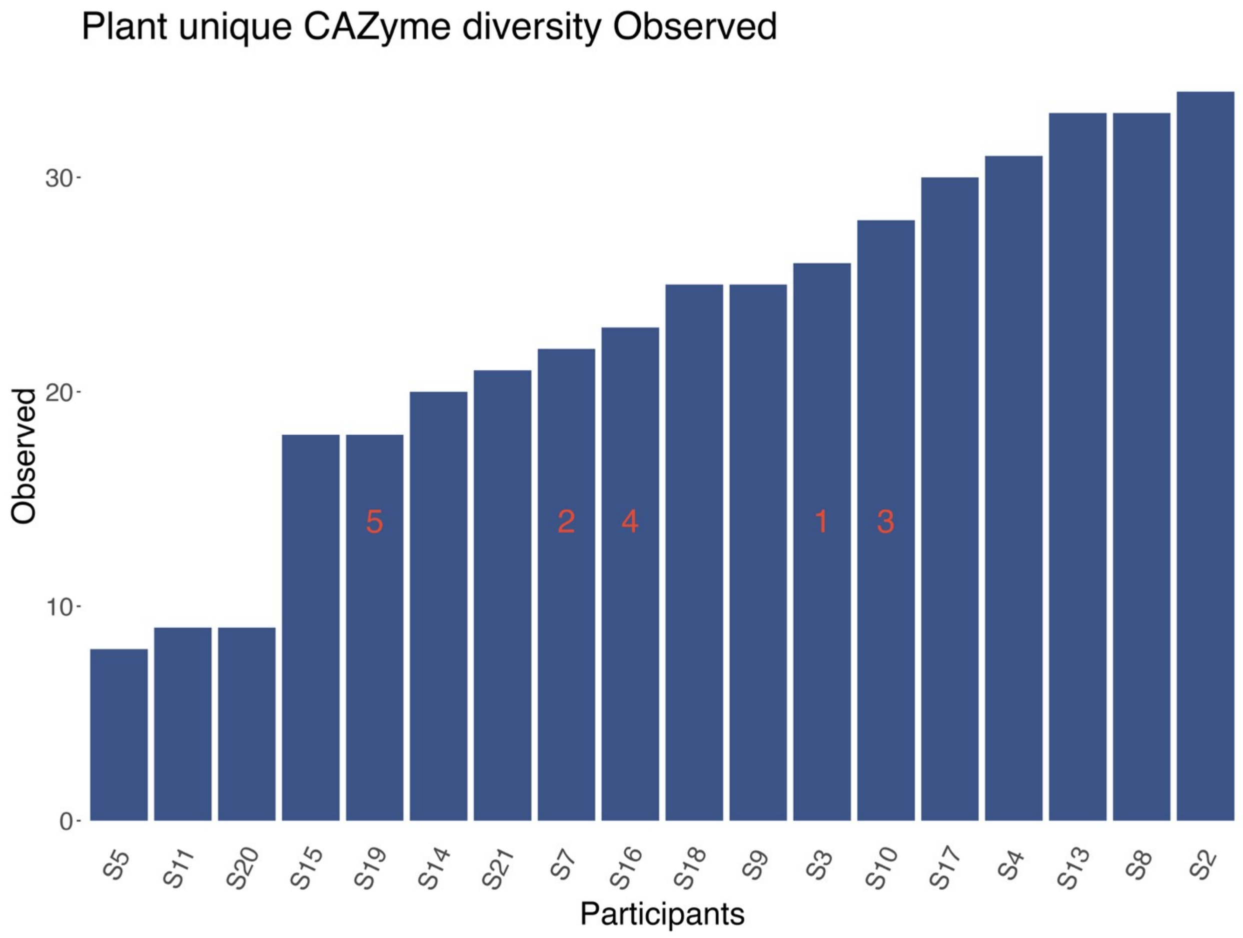

Figure S3. Observed plant unique CAZymes in the metagenomes of the 18 participants, with each bar representing one participant. The 5 chosen for the fecal fermentations are labeled with the fermentation participant IDs on the bar.

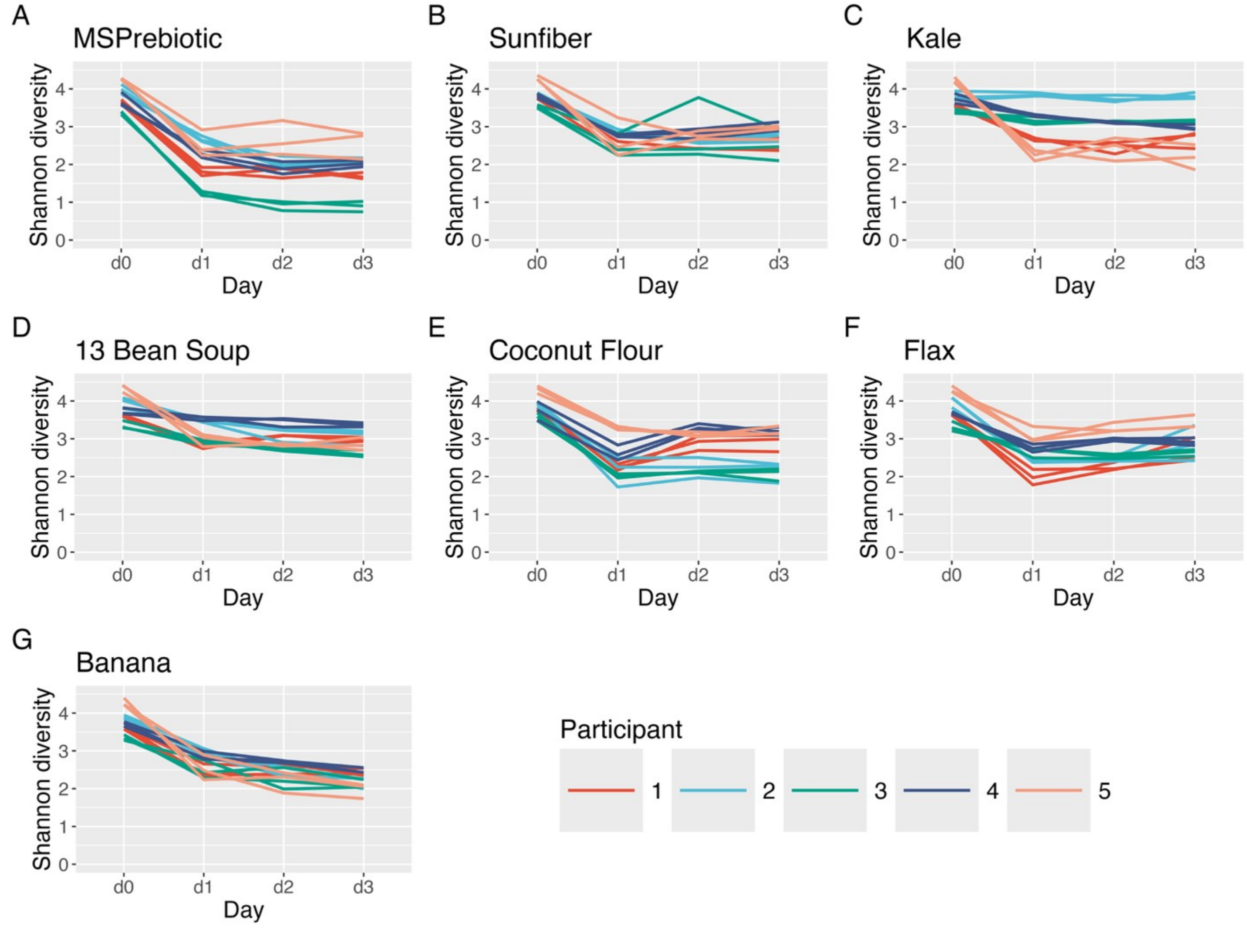

Figure S4. The change in Shannon diversity over time for each substrate

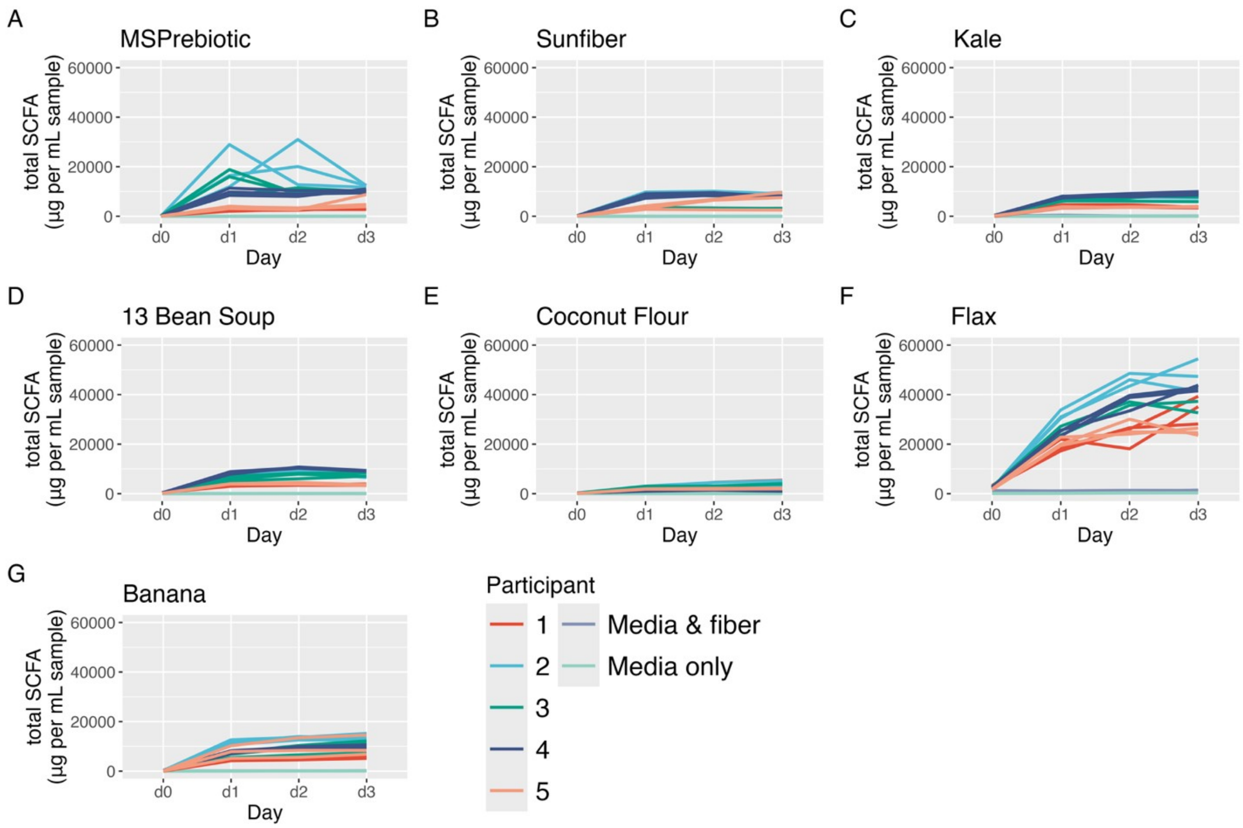

Figure S5. The change in total SCFA over time for each substrate

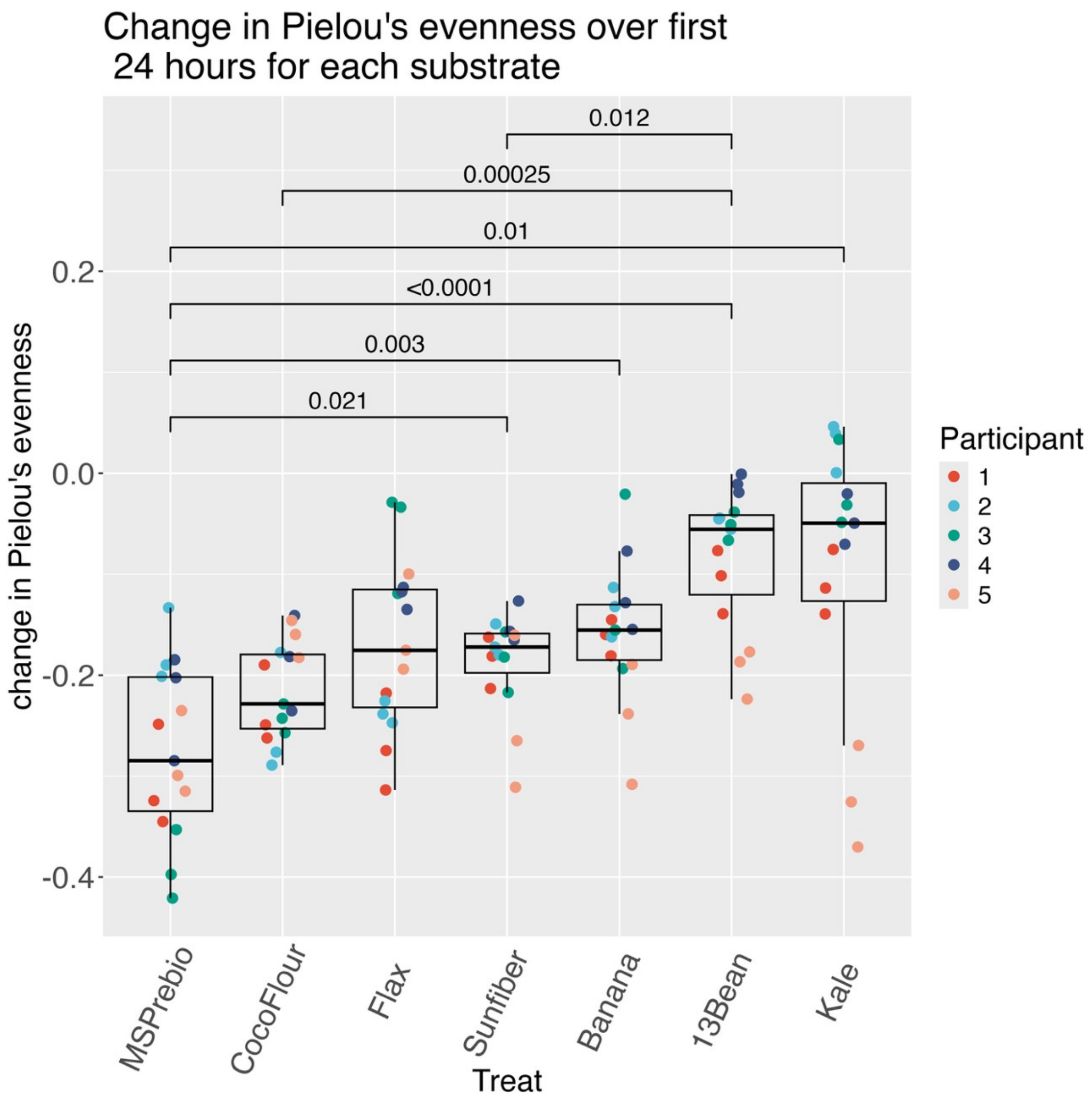

Figure S6. The change in Pielou's evenness in the first 24 hours for each substrate. Wilcoxon signed-rank tests with Bonferroni correction.

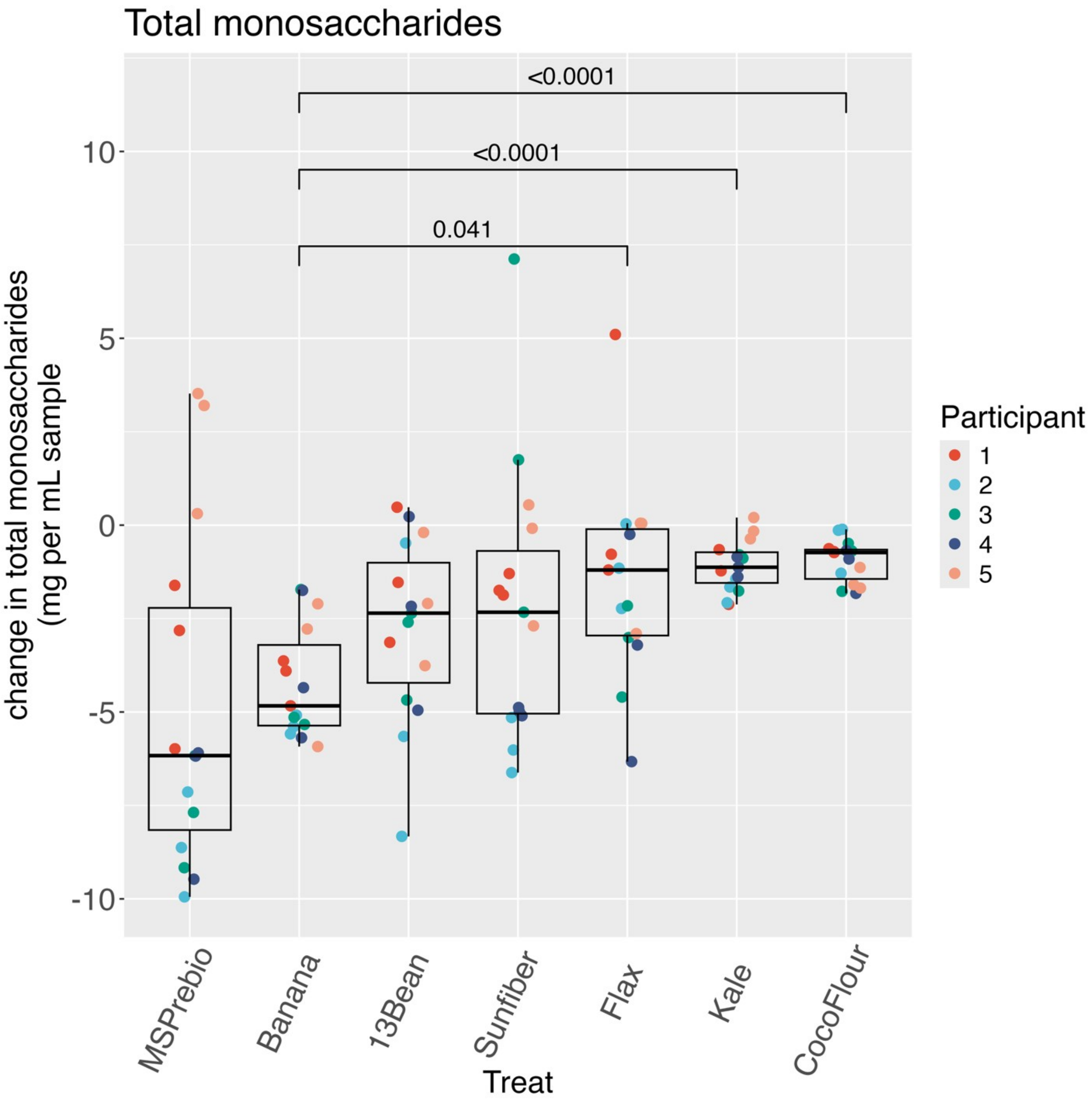

Figure S7. The change in total monosaccharides in the first 24 hours for each substrate. Wilcoxon signed-rank tests with Bonferroni correction.

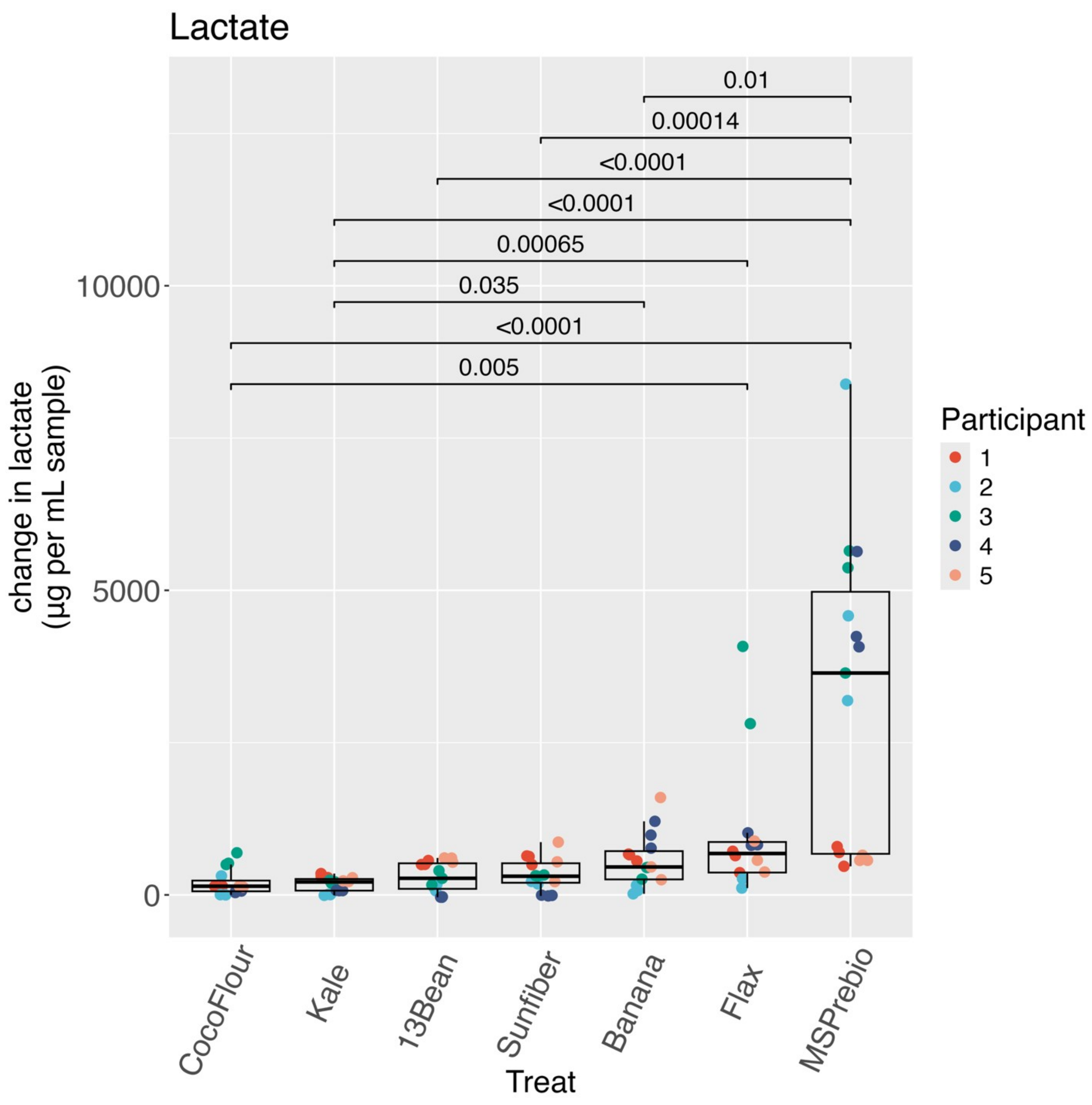

Figure S8. Changes by fiber in first 24 hours of lactate. Wilcoxon signed-rank tests with Bonferroni correction.

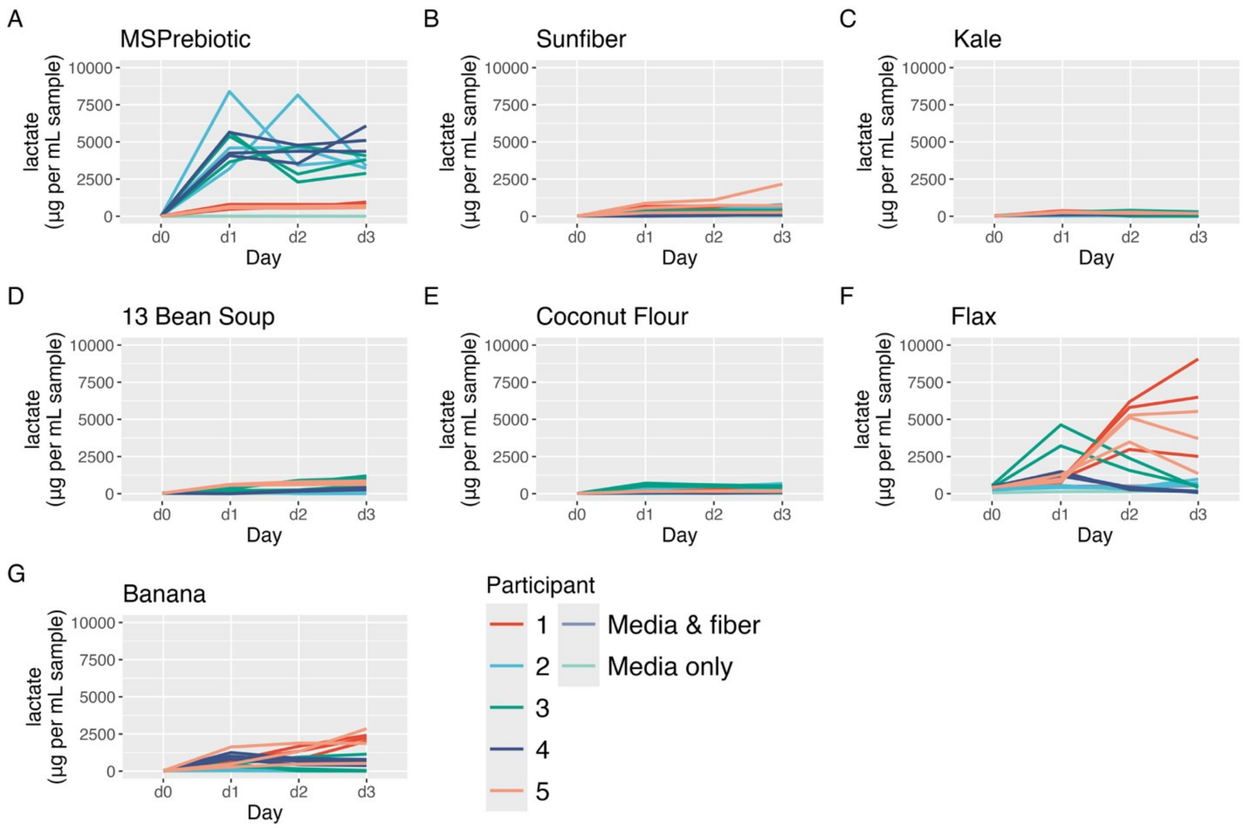

Figure S9. The change in lactate over time for each substrate

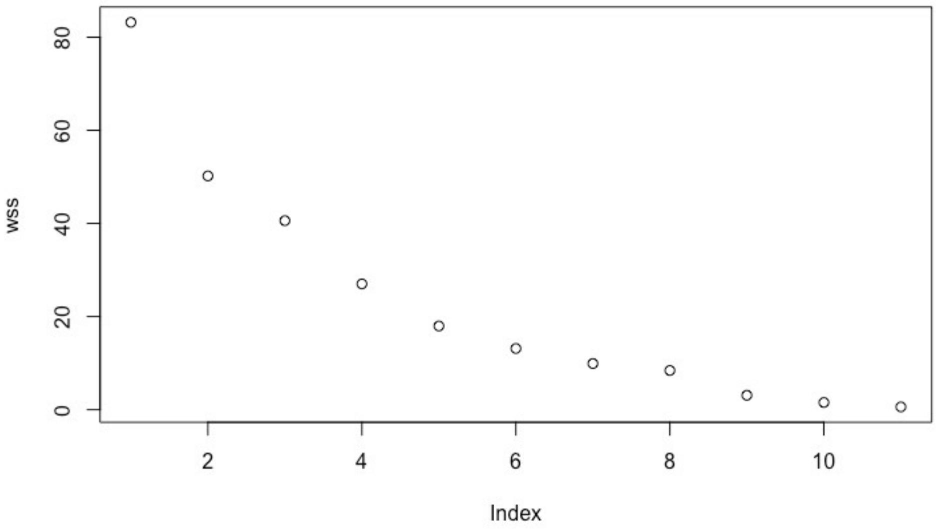

Figure S10. Plot of within cluster sum of squares (wss) for kmeans clustering in SOM

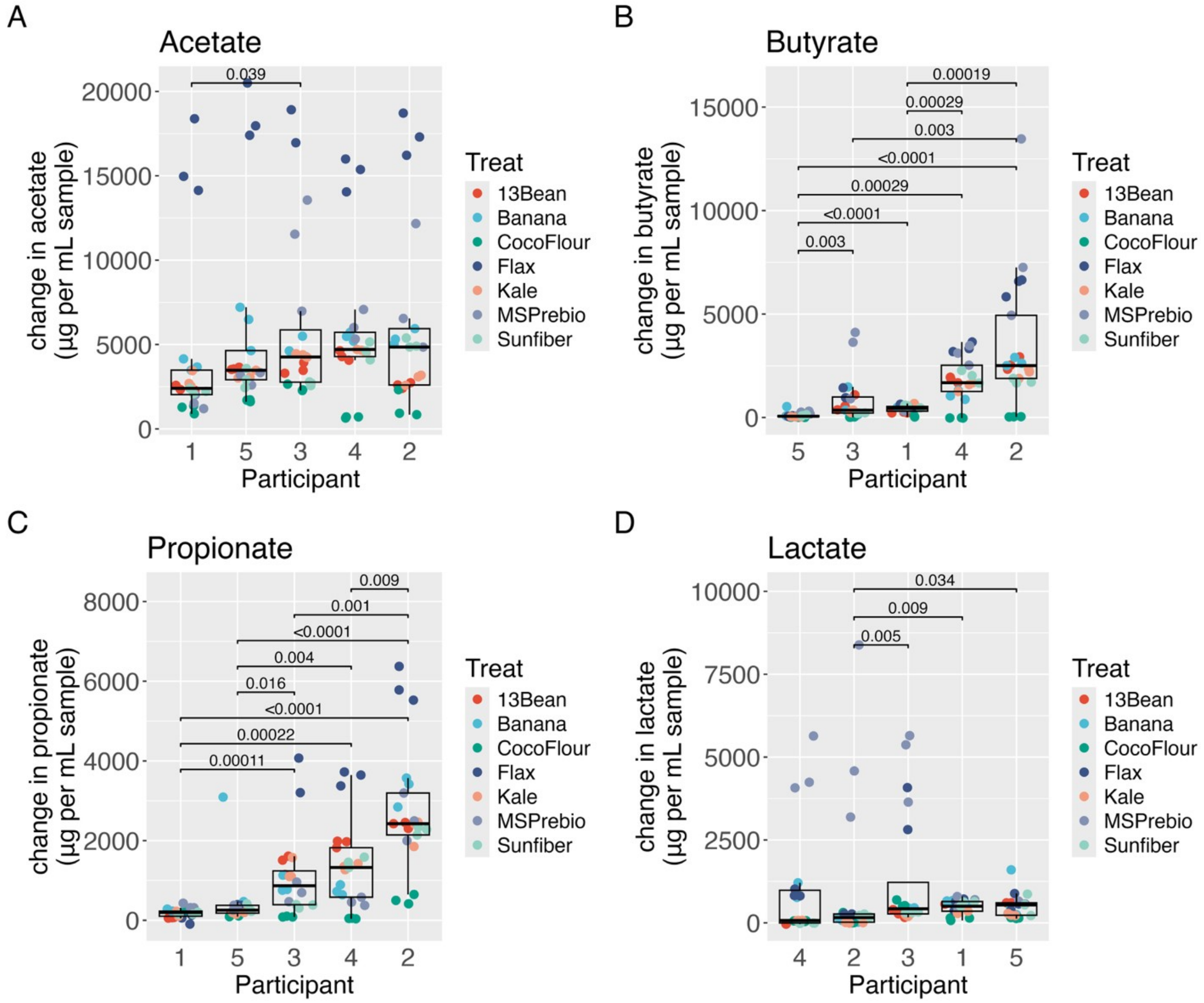

Figure S11. A. Changes by participant in first 24 hours of A. Acetate, B. Butyrate, C. Propionate, D. Lactate. Wilcoxon signed-rank tests with Bonferroni correction.
